# Forward-model-based grain reconstruction to improve the tolerance of diffraction contrast tomography for increased sample deformation

**DOI:** 10.1107/S160057672500250X

**Published:** 2025-05-12

**Authors:** Haixing Fang, Wolfgang Ludwig

**Affiliations:** a European Synchrotron Radiation Facility 71 Avenue des Martyrs Grenoble 38000 France; b Université de Lyon INSA Lyon, CNRS MATEIS Villeurbanne 69621 France; Ecole National Supérieure des Mines, Saint-Etienne, France

**Keywords:** diffraction contrast tomography, grain mapping, forward model reconstruction, synchrotron X-ray diffraction, intragranular orientation

## Abstract

To extend the applicability of synchrotron diffraction contrast tomography (DCT) towards more plastically deformed materials, we have developed a forward-model-based reconstruction method. This allows us to reconstruct grain shapes and local orientations in materials exhibiting levels of intragranular orientation spread that can no longer be handled with the conventional Friedel-pair-based indexing and tomographic reconstruction approach. This method consists of seed and regional indexing, in which an exhaustive searching and fitting of orientations is first performed to index the seed orientation, and then a regional indexing by testing a list of local orientations around the seed orientation is carried out to maximize the completeness. The capability of this novel method was benchmarked and compared with reconstructions based on the conventional Friedel-pair-matching and tomographic reconstruction method using samples made from fully recrystallized Al–Cu alloy, moderately deformed α-Ti alloy and 10% creep-ruptured Fe–Au alloy. The results show that this method has the potential to overcome the deformation constraint and can reconstruct reasonably well the intragranular orientations. It is also suitable for multi-phase reconstruction and both box-beam and line-beam acquisition geometries. The implementation has been made flexible to support the use of single or multiple GPU machines. The strengths and weaknesses of the current forward-model-based reconstruction are discussed in detail with respect to the conventional Friedel-pair-matching method. To fully exploit and complement the strengths of the two methods, the code to implement the current forward-model-based reconstruction has been fully integrated with the existing DCT code and is open source for beamline data processing.

## Abstract 2

A novel forward-model-based reconstruction method has been developed for diffraction contrast tomography and has shown great promise in increasing the tolerance of this technique for increased sample deformation. This method is suitable for multi-phase reconstruction under both box-beam and line-beam acquisition geometries and can reconstruct intragranular misorientations well.

## 1. Introduction

Diffraction contrast tomography (DCT) and near-field high-energy diffraction microscopy (nf-HEDM) are non-destructive 3D diffraction microstructure imaging (DMI) techniques to characterize grain shapes and orientations using a monochromatic X-ray beam (10–100 keV) from a synchrotron radiation source (Ludwig *et al.*, 2008 ▸; Ludwig *et al.*, 2009 ▸; Suter *et al.*, 2006 ▸). Since they were established about two decades ago, shortly after the advent of 3D X-ray diffraction (3DXRD) (Lauridsen *et al.*, 2001 ▸; Poulsen, 2004 ▸), DMI techniques have continued to evolve and constitute very powerful tools for resolving the internal structure of crystalline materials. Complementary to established electron techniques, they allow a wide range of scientific questions to be addressed, such as recrystallization, grain growth, phase transformations, plastic deformation and damage evolution, in materials including metals and alloys, ceramics, minerals, snow *etc*. (King *et al.*, 2008 ▸; Roscoat *et al.*, 2011 ▸; Zhang *et al.*, 2018 ▸; Miller *et al.*, 2020 ▸; Bhattacharya *et al.*, 2021 ▸; Zhang & Ludwig, 2024 ▸; Li *et al.*, 2024 ▸). DMI techniques are frequently combined with other synchrotron techniques such as (far-field) 3DXRD and phase contrast tomography in one instrument as well as being coupled with modeling approaches (*e.g.* crystal plasticity, dislocation dynamics and phase field) to gain a full picture of the microstructure evolution (Herbig *et al.*, 2011 ▸; Bernier *et al.*, 2020 ▸; Ravi *et al.*, 2021 ▸; Prithivirajan *et al.*, 2021 ▸; El Hachi *et al.*, 2022 ▸; Stinville *et al.*, 2023 ▸). From a technical point of view, two main variants of DMI near-field grain reconstruction strategies can be distinguished: the tomographic approach for DCT versus forward modeling for nf-HEDM.

The basic principle of the tomographic approach is based on recording diffraction spots from grains fulfilling the Bragg condition in transmission geometry by an area detector while the sample is rotating continuously around an axis perpendicular to the incident beam. To resolve the grain shape and have a sufficient two-theta angular range (typically 9–18°) to cover the reflections from the first 3 to ∼5 lattice planes, the detector normally has a small pixel size (0.5–5 µm) and has to be placed very close to the rotation axis (at distances of 2–25 mm). DCT typically uses a 2D box beam for illuminating the sample together with a semi-transparent beamstop placed directly in front of the central region of the detector to attenuate the direct beam, from which the sample shape can still be reconstructed using absorption contrast tomography (Ludwig *et al.*, 2009 ▸; Reischig *et al.*, 2013 ▸). One of the principal assets of DCT is its fast acquisition using a single rotational scan for generating a 3D grain map. In recent years, this technique has also been transformed to laboratory-based (polychromatic) DCT (LabDCT), which has triggered a massive development of grain mapping using laboratory X-rays, initially following the tomographic approach (King *et al.*, 2013 ▸; van Aarle *et al.*, 2015 ▸) and soon afterwards exploiting the forward modeling strategy (McDonald *et al.*, 2017 ▸; Bachmann *et al.*, 2019 ▸; Fang *et al.*, 2021 ▸; Fang *et al.*, 2023 ▸).

Forward modeling approaches (Suter *et al.*, 2006 ▸; Li *et al.*, 2012 ▸), the second variant, typically use a horizontally line-focused beam to illuminate the sample layer by layer. The detector is normally placed higher up to avoid background signal caused by the intense direct transmitted beam and to exploit the favorable projection geometry of high-angle reflections, yielding better grain shape discrimination. These variants, generally termed nf-HEDM, have become a common technique at synchrotron facilities such as the Advanced Photon Source and Cornell High Energy Synchrotron Source. For optimal results the data collection requires a layer-by-layer scanning procedure and may involve data collection at a second detector distance or combination with a far-field (ff) HEDM acquisition. Line-beam acquisitions are intrinsically slower compared with box-beam acquisitions and may involve non-uniform sampling (*i.e.* rendering a non-isotropic voxel size) to generate a 3D grain map. On the other hand, given the same detector configuration, line-beam illumination mode can cope with somewhat larger samples as compared with box-beam and, more importantly, the forward modeling strategy has proven to be more tolerant towards overlapping diffraction signal and can therefore cope with samples that have undergone higher levels of plastic deformation. Recently, nf-HEDM using a box beam coupled with far-field 3DXRD has also been implemented (Nygren *et al.*, 2020 ▸).

The tomographic method to reconstruct grain maps from diffraction spot data has been implemented in the DCT software package developed at the European Synchrotron Radiation Facility (ESRF) (Ludwig *et al.*, 2009 ▸; Reischig *et al.*, 2013 ▸). After image pre-processing (*i.e.* correcting detector distortion and subtracting a median filtered background) and segmentation of spots based on an adaptive threshold method, the conventional reconstruction method for DCT (gt-DCT because it originates from grain tracking) indexes the grain orientations and center-of-mass positions using Friedel-pair matching, *i.e.* identification of pairs of *hkl* and 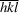 spots from the same grain that appear symmetrically after 180° rotation. Friedel pairs allow for accurate determination of scattering vectors and confine grain center-of-mass positions to lines through the illuminated sample volume. The 3D grain shapes are then reconstructed from the diffraction spots using an iterative tomographic reconstruction method (Ludwig *et al.*, 2009 ▸; Reischig *et al.*, 2013 ▸). This method is fast in reconstructing the grain map, but the reconstruction quality degrades significantly with increasing deformation fields as the reliability of the Friedel-pair-matching and indexing procedure is sensitive to diffraction spot overlaps; in addition, the distortion of the diffraction spots needs to be considered in the tomographic reconstruction problem. The introduction of a 6D-DCT reconstruction framework (Viganò *et al.*, 2016 ▸; Viganò & Ludwig, 2020 ▸) allowed the applicability of the tomographic reconstruction approach to be extended towards materials exhibiting moderate intragranular orientation spreads (1–5°). However, this method often requires extensive expertise to tune the reconstruction parameters to obtain a high-quality reconstruction.

For nf-HEDM, the *HEXOMAP* (https://github.com/HeLiuCMU/HEXOMAP; Suter *et al.*, 2006 ▸; Shen *et al.*, 2020 ▸), *HEXRD* (https://github.com/HEXRD/hexrd; Bernier *et al.*, 2011 ▸) and *MIDAS* (https://github.com/marinerhemant/MIDAS; Sharma *et al.*, 2012 ▸; Park *et al.*, 2021 ▸) programs have been developed. *HEXOMAP* is based on orientation space search and Monte Carlo optimization to perform voxel-by-voxel indexing in each 2D slice (Suter *et al.*, 2006 ▸). The code to implement this method is written with CUDA (computed unified device architecture) and runs on a GPU (graphics processing unit) computer. Effort has also recently been made to refine strain tensors for each voxel by intensity matching (Shen *et al.*, 2020 ▸). Differing from gt-DCT and *HEXOMAP*, which can work with near-field data only, *HEXRD* and *MIDAS* perform reconstructions by combining nf-HEDM and ff-HEDM, in which the indexed orientation from ff-HEDM (achieved relatively quickly because all the diffraction peaks can be assumed to originate from the sample origin and their corresponding 2θ angles can be immediately computed in far-field geometry) is fed as initial guess for nf-HEDM and a further orientation refinement around the initial guess is performed. To improve the reconstruction in a low-completeness region, an exhaustive orientation search is also carried out (Bernier *et al.*, 2020 ▸; Nygren *et al.*, 2020 ▸).

**Figure 1:**
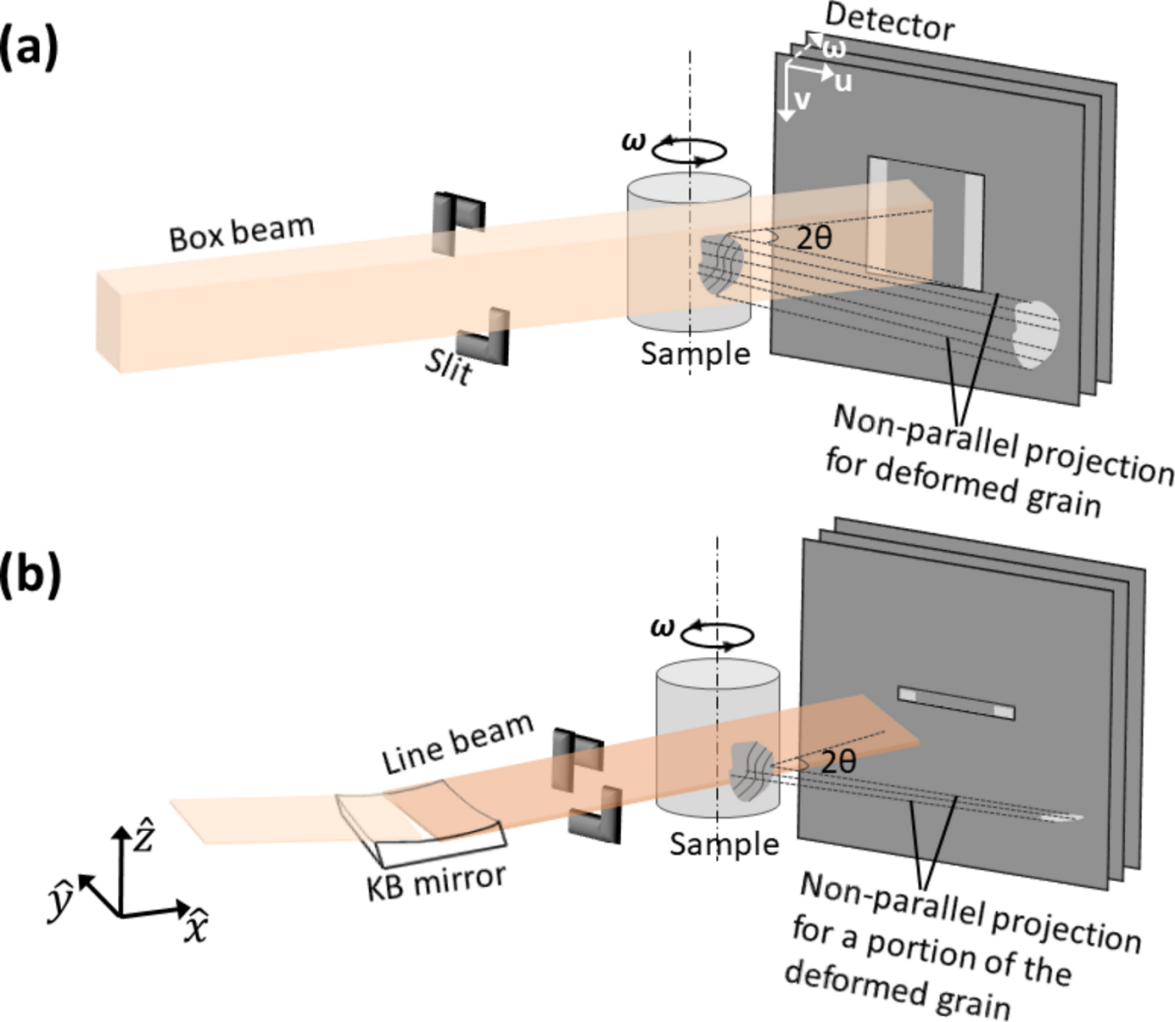
Schematic diagram of (*a*) box-beam and (*b*) line-beam DCT. In box-beam DCT, the beam size is defined by the slit and the illuminated sample volume can be considered as 3D, while the beam is focused to a line shape by Kirkpatrick–Baez (KB) mirrors and illuminates a 2D section of the sample in the line-beam acquisition. The laboratory coordinate system is right handed and defined as follows: 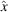 is along the X-ray beam, 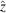 is along the rotation axis, and 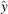 is transverse and perpendicular to the plane defined by 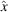 and 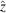. The detector system is defined as follows: the origin sits on the top-left corner, *u* is horizontal and perpendicular to the beam, *v* points down, and ω is along the rotation direction.

As a full-field technique in the near-field geometry, DCT faces a serious constraint when applied to deformed microstructures, from which the diffraction spots are distorted (due to local changes in the direction of the scattering vector in different regions of the same grain) and spread in the detector plane and across the rotation angles (see Fig. 1 ▸), leading to a highly heterogeneous intensity distribution within the dif­frac­tion blobs and broadening in azimuthal and omega angles. This poses big challenges for 6D-DCT to properly reconstruct deformed grains with strains larger than a few per cent. To overcome the deformation constraint and perform the grain reconstruction efficiently and autonomously without the need to acquire strong expertise in the technique, we have developed a forward-model-based reconstruction method for both box-beam and line-beam DCT, referred to as fwd-DCT. This method was first benchmarked on a fully recrystallized Al–Cu alloy sample to characterize its performance for multi-phase reconstruction and compared with gt-DCT in detail. Then, fwd-DCT was applied to reconstruct grain maps for a moderately deformed α-Ti alloy and a highly deformed Fe–Au alloy sample to demonstrate its capabilities in overcoming the deformation constraint. Notably, the quality of the grain maps, characterized by *e.g.* grain-boundary positions and intragranular orientations, can be validated by either phase contrast tomography (Groso *et al.*, 2006 ▸) or other reconstructions and simulations [*e.g.* 6D-DCT reconstruction for moderately deformed materials and comparison between forward-projected spots and the experimental ones (Viganò *et al.*, 2016 ▸; Viganò & Ludwig, 2020 ▸)]. Like all other nf-HEDM forward modeling approaches (although *HEXOMAP* also uses intensity matching for strain optimization), the current fwd-DCT method works on binarized spots, whereas gt-DCT reconstruction takes into account diffracted intensities for 3D shape and 6D orientation reconstructions. Both approaches have distinct advantages and limitations, and combining these strategies can be beneficial. Therefore, to complement the advantages of gt-DCT and fwd-DCT, the code to implement fwd-DCT has been fully integrated with the existing code for gt-DCT to expand the application of DCT to a wider range of materials and facilitate autonomous grain reconstruction without the need for strong expertise in this technique.

## 2. Forward-model-based reconstruction

### 2.1. Reconstruction principle and algorithm

The fwd-DCT reconstruction is based on a kinematic forward-projection model to index orientations of each voxel within the sample volume, thereby reconstructing the grain shapes and intragranular orientations. The core principle is to find the orientation that maximizes the completeness value for a given point within the sample volume. As presented in many other forward models (*e.g.* Suter *et al.*, 2006 ▸; Bernier *et al.*, 2011 ▸; Bachmann *et al.*, 2019 ▸; Fang *et al.*, 2022 ▸), the completeness is defined as the ratio of the number of intersected spots between the forward projection and experimentally detected spots to the number of theoretically expected spots on the detector. Notably, the current fwd-DCT method is developed for the case of a monochromatic parallel beam [see Fang *et al.* (2023 ▸) and Oji *et al.* (2024 ▸) for white-beam DCT reconstruction using laboratory X-rays and thermal neutrons, respectively] and tries to reconstruct the orientation for each voxel. Unlike the tomographic method, which estimates the grain average elastic strain and applies a small correction to the projection geometry of the individual spots (Reischig & Ludwig, 2020 ▸), the voxel-based forward model in this work does not currently include this contribution. However, in the presence of large lattice rotations, elastic strains can be considered as minor perturbations especially for isotropic lattice structure. The details of the forward projection are presented in Appendix *A*[App appa].

**Figure 2:**
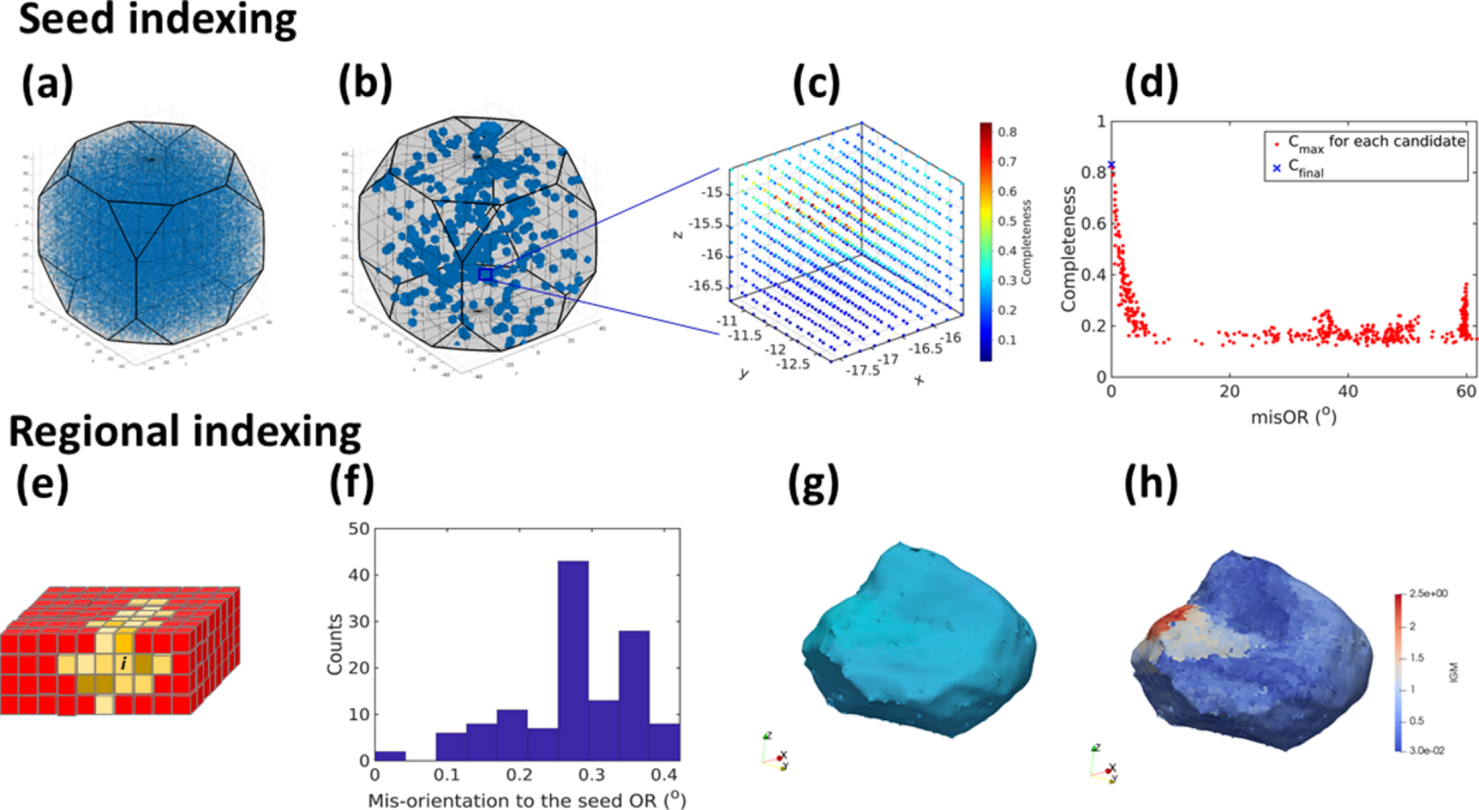
Seed (top row) and regional (bottom row) indexing for fwd-DCT illustrated by an example for indexing orientations in a cubic crystal. (*a*) The fundamental orientation space is discretized to 262144 orientations with an average distance of 2°; (*b*) plot of locally sampled orientations (*N*_candidate_ × *N*_local_OR_ = 500 × 1001 = 500500) in axis–angle space with one clustered cloud representing one local orientation set as shown by a zoomed-in example in (*c*) colored by the corresponding completeness; (*d*) plot of *C*_max_ within each local orientation set as a function of the misorientation angle with respect to the final indexed orientation, which ultimately gives a final highest completeness *C*_final_ = 0.83; (*e*) a 3D region containing the seeding voxel *i* showing accepted (colored by different degrees of yellow representing different local orientations) and rejected (colored red) indexing using the orientations around the seed orientation; (*f*) histogram of the misorientations between the list of 126 orientations and the seeding orientation with a maximum distance of 0.4° and a mean distance of 0.27°; (*g*) a reconstructed iron grain colored by inverse pole figure along *Z* (IPF-*Z*) and (*h*) its intragranular misorientations showing higher misorientations on the left side.

The current fwd-DCT reconstruction process consists of two main steps: (i) index orientation of a seeding voxel and (ii) index orientations of regional voxels surrounding the seeding voxel. Fig. 2 ▸ shows an example to illustrate the algorithm for each step. To index the seeding voxel *i*, it starts with searching for candidates in the global orientation space followed by refining and fitting local orientations to find the solution *U_i_* yielding maximum completeness. As shown in Fig. 2 ▸(*a*), the fundamental zone of the orientation space is first discretized to have a uniform distance (usually 1–3° for *N*_global_OR_ orientations) for a given crystal structure. Then, forward calculations are performed for each of the discretized orientations and the orientations are ranked according to their forward-calculated completeness values. At this stage, most orientations generate very low completeness (<0.1) and even the highest completeness value is still rather low (<0.3 in most cases). Subsequently, the top-ranking orientations with the highest completeness values are selected as candidates (*e.g.N*_candidate_ = 500) for local sampling, resulting in a set of orientations (usually *N*_local_OR_ = 1001) distributed around each candidate orientation with a maximum distance of ∼1.8° and a mean distance of ∼1° [Figs. 2 ▸(*b*) and 2 ▸(*c*)]. A further forward calculation is performed on all the locally sampled orientations, *i.e. N*_candidate_ × *N*_local_OR_ orientations, from which a unique orientation yielding the maximum completeness (*C*_max_) within its own local orientation set can be identified. Finally, the most outstanding orientation rendering *C*_max_ among all the tested orientations is used as the starting value for a least-squares fitting to minimize the objective, obj = 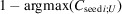, resulting in the final solution *U_i_* for the orientation out of all candidates with small misorientation angles as well as with twin relationships [Fig. 2 ▸(*d*)]. When the seed completeness, *C*(seed *i*; *U_i_*), is larger than both the pre-set minimum completeness value *C*_min_ and its existing completeness value (= 0 at the start of the reconstruction), the seed indexing result is considered as successful and the seed orientation is updated. Although some redundant computations for similar orientations may be performed with this local sampling, particularly for seeding points within the same deformed grains, this algorithm ensures successful indexing and does not require tuning of reconstruction parameters for different samples, thereby facilitating an autonomous reconstruction without expert user intervention.

Once the seed indexing is successful, a region of voxels (*N*_region_voxel_ voxels) surrounding the seed *i* is identified by estimating the size of a 3D bounding box from the sizes of the corresponding diffraction spots [Fig. 2 ▸(*e*)]. Then, a list of orientations around *U_i_* (*N*_region_OR_ orientations) is generated [Fig. 2 ▸(*f*)] and used to perform forward calculation for each of the voxels *j* in this region to determine its maximum completeness value, *C*(voxel *j*; *U_j_*). When 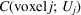 > 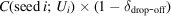, with δ_drop-off_ a pre-defined value (usually 0.02–0.05), and *C*(voxel *j*; *U_j_*) is larger than its existing value, *U_j_* is accepted as the new orientation for the voxel *j* [yellow voxels in Fig. 2 ▸(*e*)]; otherwise, the indexing is rejected [red voxels in Fig. 2 ▸(*e*)]. This not only allows the regional voxels to vary their orientations locally to maximize their completeness values but also enables parallel computation without the need to check the spatial connectivity between the voxels so that the calculation can run in parallel for all the voxels in a given region on a GPU. Therefore, a meaningful reconstruction of local orientations can be achieved in a computationally efficient manner. Notably, the regional indexing is automatically controlled by the completeness drop-off value. This means that local orientations resulting in completeness differences larger than the corresponding drop-off will be excluded in one regional indexing but could be reconstructed in another one. In general, this results in a faster reconstruction for samples in a less deformed state than for those in a more highly deformed state.

When the regional indexing is finished, a completeness-weighted center position is identified and the distance to the original seeding position is calculated. If the distance is smaller than a pre-defined tolerance (typically 3 pixels), the whole run for this seeding point is considered as done; otherwise, it will continue by taking the weighted center as a new seeding position for further seed and regional indexing, in which the forward calculations with global orientations are not required and local orientation sampling is only done around *U_i_*, whilst the rest of the processing remains the same as described above.

By repeating this seed and regional indexing procedure for different seeding points, grain shapes and local orientations can be reconstructed. Notably, an iterative procedure to generate seeding points is used. During each iteration, the number of seeding points is first calculated by dividing a volume determined by the total number of candidate voxels (*i.e.* initially the whole sample volume) by an equivalent cubic volume (with decreasing edge length *L*_side_, starting from 45 pixels in the first iteration down to 3 pixels). Then, the seeding points are placed at the locations at a distance from the already-reconstructed voxels with completeness values higher than a trust completeness (= 0.95) based on a computed distance map. In addition, the distance between any two seeding points should be larger than a minimum allowed value (determined by half of *L*_side_) and not smaller than 3 pixels at each iteration. This distance is then used to sort the priorities for assigning voxels to be seeding points, *i.e.* the voxel with a bigger distance has a higher priority to become a seeding point. In this way, the number and the distribution of the seeding points are optimized for speeding up a good grain reconstruction (further details are given in Section 2.2). Fig. 2 ▸(*g*) shows a reconstructed deformed grain (from the Fe–Au sample described in Section 3.1) with significant intragranular misorientations (IGM), for which the grain surface in contact with a grain-boundary crack has much higher misorientation (∼2° with respect to the mean orientation) than the rest of the grain [Fig. 2 ▸(*h*)].

### 2.2. Implementation

During each seed and regional indexing, a large amount of forward calculation is required for (i) identifying orientation candidates from global discretized orientations; (ii) indexing the final orientation for the seeding voxel by refining over locally sampled orientations; and (iii) indexing regional voxels by checking completeness values for a list of local orientations around the seeding orientation. The number of forward calculations for these three procedures is *N*_global_OR_ (see Table 1 ▸ for the values for different crystal structures), *N*_candidate_ × *N*_local_OR_ and *N*_region_voxel_ × *N*_region_OR_, respectively, which are all in the order of hundreds of thousands of calculations. To perform such calculations realistically and efficiently, CUDA-based programming with a modern GPU has been implemented and benchmarked as taking ∼2 s for each of the three procedures with an NVIDIA A40 48 GB GPU.

**Figure 3:**
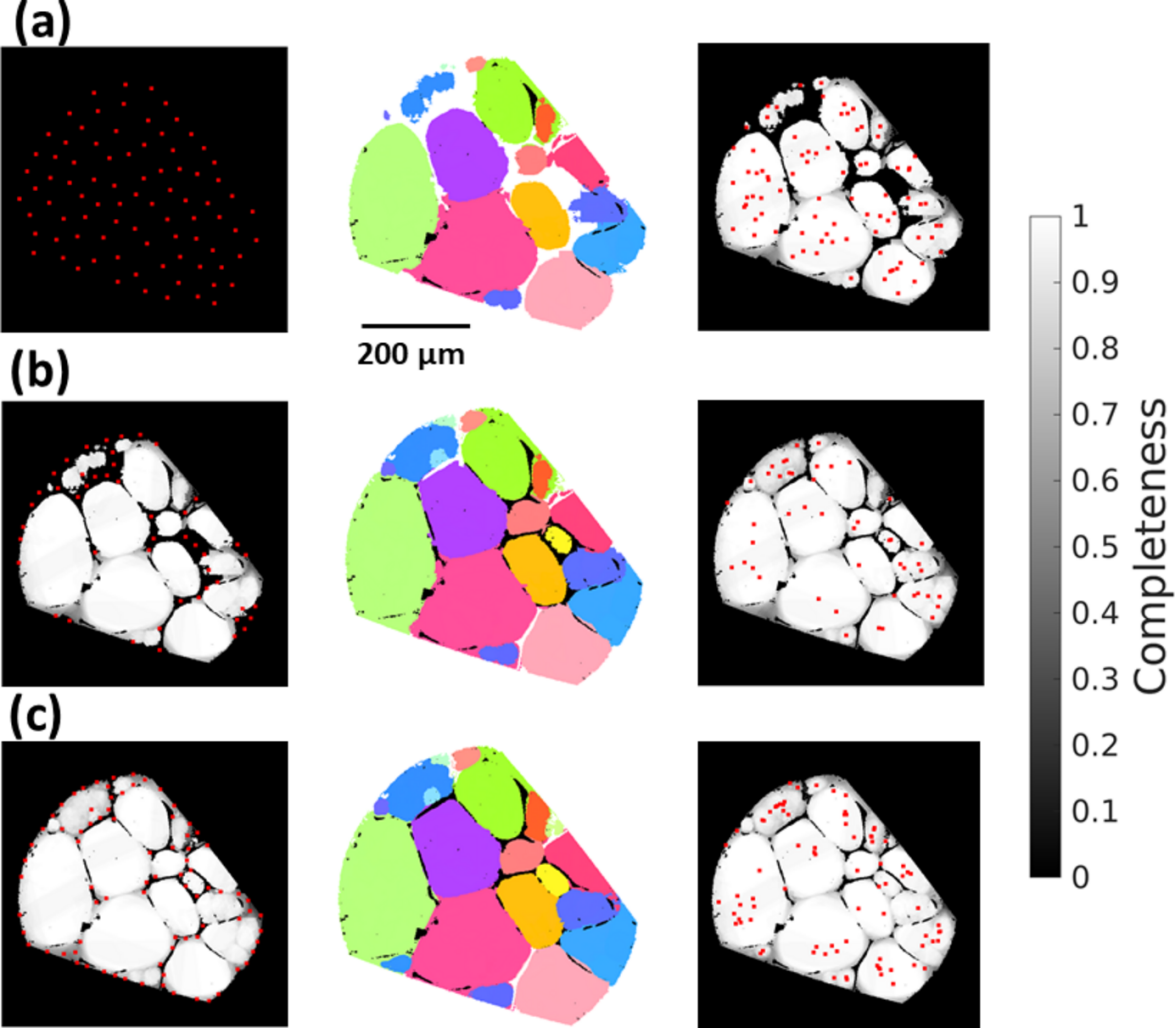
A slice reconstruction showing the positions of the seeding points (marked by red points) at the start of each iteration plotted on the completeness map from the last reconstruction (left), the resulting reconstructed grain map (middle) and the updated positions of the seeding points on the completeness map after the reconstruction (right) for a successive iteration of (*a*) 1, (*b*) 2 and (*c*) 3, which contain 85, 48 and 82 seeding points, respectively.

To reconstruct the whole grain map, an iterative reconstruction is employed for different seeding points generated with different gridding levels of the sample volume. At each iteration, the locations of the seeding points are prioritized as those with lowest completeness values, whilst their inter-distances must be greater than a minimum distance determined by the gridding level, which decreases with increasing the number of iterations (see Section 2.1). Fig. 3 ▸ illustrates the iterative reconstruction for a single slice reconstruction. In the first iteration, the seeding points are generated directly on the sample mask, *i.e.* a zero-completeness map. After all the seeding points and the corresponding regions are indexed, most of the sample is reconstructed and the updated positions of the seeding points can be seen as distributed close to the grain centroids because of the iterative replacement by the completeness-weighted centers. In the second iteration, the seeding points are mainly generated on either the empty indexed or low-completeness regions, which have priorities over the positions in the high-completeness regions. As a result, the reconstruction almost fills up the whole sample mask. In the third iteration, a new set of seeding points is generated in low-completeness regions, *i.e.* mainly around grain boundaries, and the subsequent indexing identifies small grains and refines the grain shape reconstruction. Notably, the iterative seed and regional indexing, in particular on the low-completeness regions, is the key to achieving a high-quality grain map for fwd-DCT. In general, the more seeding points are tested, the better the grain map will be.

To limit the reconstruction to the sample volume, a sample mask is usually segmented from absorption or phase contrast tomography where the sample position remains the same as in the DCT scan. For multi-phase materials, the corresponding multi-phase grain mapping can be readily achieved using different volume masks when the contrast is sufficient to identify different phases. The voxel size of the grain reconstruction is set to match that of the tomography volume, which normally has the same size as the effective pixel size of the detector but can be augmented according to different requirements. The fwd-DCT reconstruction can be done on one computer equipped with one GPU card for volumetric reconstruction at once or on multiple computers, each with a GPU card, for slice-by-slice reconstruction. The latter has the advantage of being faster when the GPU resources are sufficient. The reconstruction stops when the maximum number of iterations is finished. This corresponds to typically 10 iterations for volumetric reconstruction and 5 iterations for slice-by-slice reconstruction. Further iterations or choosing particular seeding points is sometimes required to improve the grain reconstruction.

When the reconstruction is finished, a grain map is produced by merging regions with misorientations smaller than a pre-defined value (typically 3°), whilst each voxel orientation remains intact. Optional manipulations on the grain map include checking the reconstruction of small grains, cleaning noise and filtering low-completeness voxels grain by grain according to their normalized completeness [voxel completeness divided by the maximum within the same grain (Hayashi & Kimura, 2023 ▸)]. The final exported grain map is compatible with the LabDCT data format (Bachmann *et al.*, 2019 ▸; Fang *et al.*, 2023 ▸) and can be easily visualized in *ParaView* (https://www.paraview.org/). Following Fang *et al.* (2020 ▸), a forward simulation model is also developed to compute spot intensities diffracted from every voxel in a given grain map at any rotation angle by considering sample structure factor, attenuation, Lorentz and polarization effects, and detector point spread. Such a forward simulation model is useful to verify the quality of the reconstructed grain map by comparing the simulated projections with the experimental ones.

### 2.3. Code availability and integration with gt-DCT

The fwd-DCT code is written in MATLAB and CUDA and is open source (https://gitlab.esrf.fr/graintracking/dct/-/tree/new_diffractometer?ref_type=heads). It has been deployed for beamline user data processing since August 2023 and is fully integrated with the main gt-DCT code, facilitating the combined use of the two different methods for improving grain mapping. For example, fwd-DCT can be applied to find missing grains for the gt-DCT grain map and foster the 6D-DCT reconstruction (Viganò *et al.*, 2016 ▸) with the new indexed grains.

In contrast to other forward-model-based reconstruction methods for near-field grain mapping (*e.g.* Nygren *et al.*, 2020 ▸; *MIDAS* software for HEDM), the current fwd-DCT method does not require data collection with multiple detector distances (for fitting the geometry or to locate the grain center of mass) or rely on far-field indexing for seeding the orientations as known *a priori*. It is suitable for both box-beam and line-beam acquisitions (see Fig. 1 ▸) at one single distance, whilst the geometry is either self-fitted via a pre-reconstructed grain map, which is then used to forward-project spots and compare with experimental ones, or directly obtained from the Friedel-pair matching with gt-DCT (Ludwig *et al.*, 2009 ▸).

## 3. Experiment

### 3.1. Samples

Three types of samples were selected to demonstrate different aspects of the present fwd-DCT method: (i) a recrystallized multi-phase AlCu alloy sample with grain boundaries decorated by an intermetallic phase to showcase the multi-phase reconstruction with fwd-DCT, assess the fidelity of fwd-DCT reconstruction and compare with gt-DCT reconstruction; (ii) a moderately deformed α-Ti single-phase sample measured by both box-beam and line-beam DCT to highlight the versatility of fwd-DCT and the differences in the grain maps resulting from different beam fluxes and shapes; (iii) an Fe–Au alloy sample ruptured after creep testing to benchmark the capability of fwd-DCT reconstruction for even more deformed samples.

The AlCu alloy (8 wt% Cu) sample was polished to a wedge shape (width × thickness × height ≃ 600 × 450 × 1000 µm) after wire-cutting from a heat-treated cylinder rod with a diameter of ∼3 mm and a length of ∼6 mm. The rod was first annealed at 580 °C for 1 h and then underwent slow cooling in a furnace at an initial cooling rate of ∼3.5 °C min^−1^ with the intention of removing any lattice strains. During annealing, the sample was in a solid–liquid two-phase region with an equilibrium solid fraction of 83.3% according to the lever rule calculation on the Al–Cu phase diagram. During cooling, a Cu-enriched eutectic phase (known as Al_2_Cu θ phase with a tetragonal lattice) solidified and formed a layer of crystallites (with sizes ranging from a few micrometres to nanometres) at grain boundaries of the Al matrix. Therefore, the grain shapes of the Al matrix can be resolved by phase or absorption contrast tomography due to the significant contrast between the θ phase and the face-centered cubic Al. More details of the sample preparation can be found elsewhere (Fang *et al.*, 2023 ▸).

The α-Ti sample with a hexagonal close-packed structure was in a dog-bone shape machined from a heat-treated commercially pure Ti alloy. The sample was tensile-loaded to ∼2% plastic deformation using the Nanox miniature stress rig (Gueninchault *et al.*, 2016 ▸). To facilitate the selection of the sample field of view with the line beam, a ∼30 µm cube was attached on the sample surface and served as a marker for regional selection. Notably, this sample has a typical cold-rolled Ti texture (Keeler & Geisler, 1956 ▸). More details about this sample have been reported by Ribart *et al.* (2023 ▸).

The Fe–Au sample has a cross section of ∼250 × 250 µm and a length of ∼1 mm. It was cut by spark-erosion from a dog-bone-shaped creep-failed sample, which was slowly deformed at 550 °C and 80 MPa for 376 h until rupture at a macroscopic strain of 10% (Fang *et al.*, 2016 ▸). This sample has served as a demonstration of a self-healing alloy where the initially dissolved supersaturated gold atoms in the matrix could diffuse to the grain-boundary cavities and form Au-rich precipitates therein (Fang *et al.*, 2016 ▸; Fu *et al.*, 2022 ▸). Therefore, the grain shapes of the iron matrix with a body-centered cubic structure can also be delineated by grain-boundary phases (*i.e.* cavities or precipitates), which are useful for validating the fwd-DCT reconstruction for deformed samples.

### 3.2. Data collection for synchrotron DCT

Synchrotron DCT experiments were performed on beamline ID11 at the ESRF. A parallel monochromatic X-ray beam with an energy of 43.56 keV was used to illuminate the samples. A sCMOS (Andor Marana) detector with 2048 × 2048 pixels was placed behind the sample at a distance of 5–10 mm from the vertical rotation axis, which is perpendicular to the X-ray beam. Diffraction signals were recorded by the outer area of the detector, while the transmitted direct beam was attenuated by a tungsten beamstop (100–200 µm thickness) and recorded by the central area of the detector. The detector can be coupled to objective lenses with different magnifications, resulting in an effective pixel size of 1–2 µm. For each DCT scan, a series of 3600 or 7200 equally spaced projections were acquired during continuous sample rotation over 360° with an exposure time (*t*_exp_) of 0.05–0.1 s for each projection. The X-ray beam size, sample-to-detector distance (*L*_sd_), detector objective lens, beamstop size and thickness, and exposure time were adjusted according to different sample characters and dimensions, to enhance the diffraction signals as much as possible, whilst not saturating the detector, in particular with the transmitted direct beam. To measure samples with a large vertical dimension resulting in serious overlapping of diffraction spots, multiple vertical DCT scans for different sample regions were performed with an overlapped area (overlapping dimension usually corresponding to half of the average grain size) between adjacent scans.

Table 2 ▸ gives details of the DCT scan parameters for each sample. All samples were measured with box-beam DCT [see Fig. 1 ▸(*a*)], while a line-beam DCT scan was also performed for the α-Ti sample. The line beam was achieved using KB mirrors to vertically focus the beam producing a full width at half-maximum (FWHM) of ∼2 µm [see Fig. 1 ▸(*b*)], while the full vertical size for the sample illumination was ∼8 µm due to the beam tails. With the line focusing, an increase of about 28 times in the flux at the sample position was observed, and this resulted in about 63 times more photons per unit sample volume and 1.8 times better contrast-to-noise ratio (CNR, defined as the difference in the intensities between the spot and the background divided by the standard deviation of the background intensity) for the DCT measurements of the Ti sample here (CNR ≃ 36 for the line beam and CNR ≃ 20 for the box beam). Notably, line-beam DCT has not yet become a routine measurement at ID11 as the current line-focusing optics deflect the X-ray beam from the horizontal plane; thus, the diffractometer has to be adapted and inclined to ensure the rotation axis is perpendicular to the beam. Whilst such instrument alignment is feasible, it is not ideal for automated operation of the instrument at present. A new set of line-focusing optics is expected to be installed to create better conditions for the line-beam DCT measurements in the near future. Here we focus on demonstrating the capability of fwd-DCT for reconstructing the line-beam DCT data.

### 3.3. Grain reconstruction and data analysis

The currently developed fwd-DCT method was used to reconstruct all the grain maps. As it is integrated with the gt-DCT code, it shares the same pre-processing steps, *i.e.* correction of detector image distortion and rolling median background subtraction. After the pre-processing, a Laplacian-of-Gaussian (LoG) based method was employed to segment the diffraction spots and create images containing spots only (Lind, 2013 ▸; Fang *et al.*, 2021 ▸). Although fwd-DCT can pre-reconstruct a grain map, and use the reconstructed grains to forward project spots and minimize their differences with the experimental ones to optimize the geometry, we directly used the fitted geometry from the Friedel-pair matching. To provide a sample mask for the fwd-DCT reconstruction, a tomography volume was reconstructed using the *ASTRA* toolbox (van Aarle *et al.*, 2016 ▸) from the flat-field-corrected images that were cropped from the central area of the detector. Then, the absorption volume was segmented to identify different phases, *e.g.* matrix, cavities or precipitates, and provide volume masks for fwd-DCT reconstruction.

Grain reconstructions with fwd-DCT were launched as remote tasks to GPU machines on the ESRF computing cluster once the diffraction spots were segmented, the volume mask was obtained and the geometry was optimized for each dataset. The reconstruction parameters were all set to be the same with a minimum completeness of *C*_min_ = 0.2 and a drop-off of δ_drop-off_ = 0.02. For grain identification, a misorientation of 3° was selected for the un-deformed sample (*i.e.* the AlCu alloy sample) and 4° was used for deformed samples (*i.e.* the α-Ti sample and the Fe–Au sample). For the AlCu and α-Ti samples, we also employed the gt-DCT method with the 6D reconstruction algorithm (Viganò *et al.*, 2016 ▸; Viganò & Ludwig, 2020 ▸) to reconstruct the grain map and compare with the result from fwd-DCT.

After the grain map was obtained, a series of analyses were performed with different utilities within the DCT code package. These analyses include manipulating the grain maps (*e.g.* cropping, shifting, rotating and stitching), comparing different grain maps, extracting grain boundaries and registering with tomography volumes *etc*. For visualization, the grains are colored by IPF-*Z* in the sample coordinate system.

## 4. Results

### 4.1. Reconstruction of the Al–Cu alloy sample and comparison with gt-DCT

**Figure 4:**
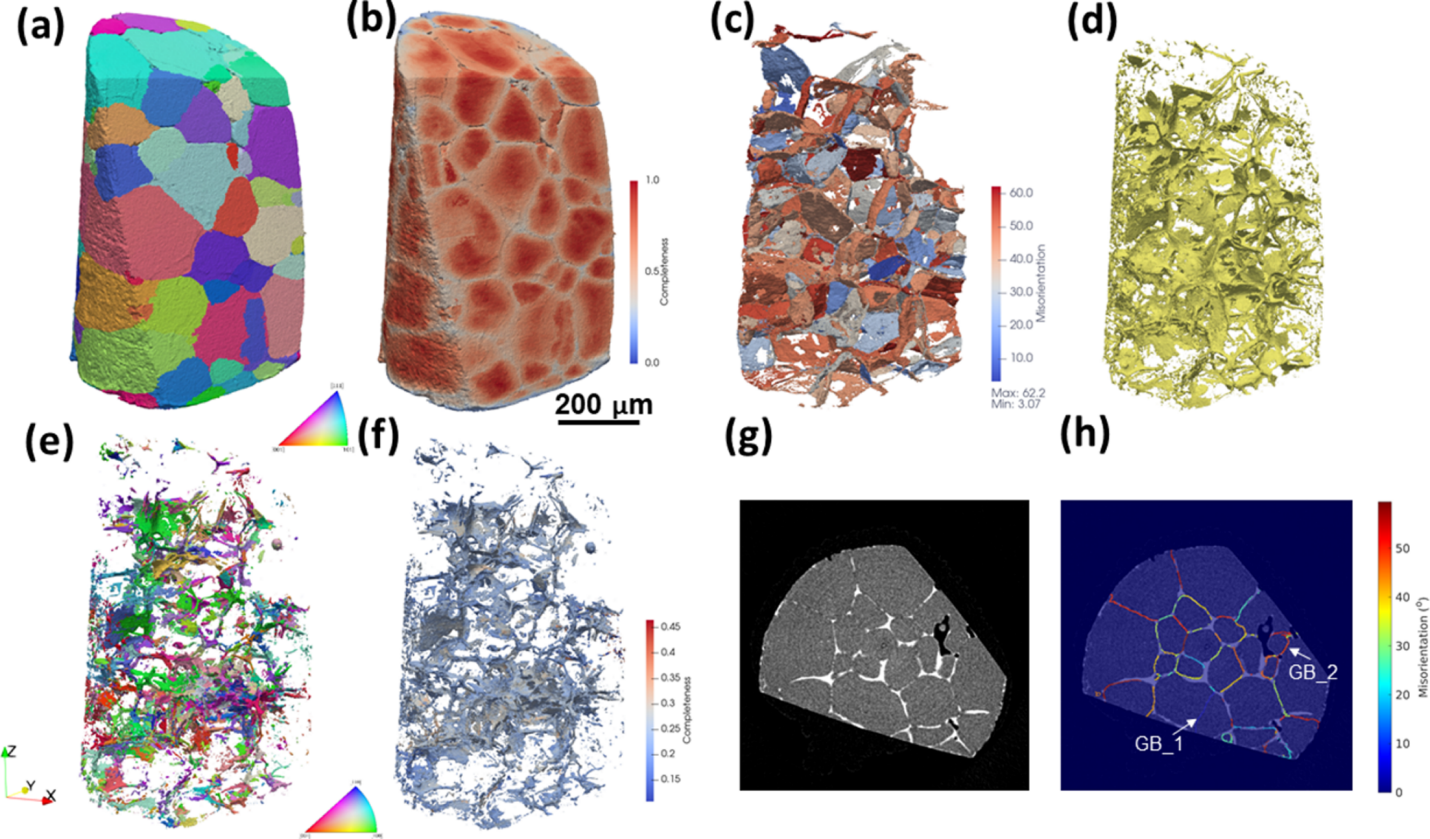
Multi-phase grain reconstruction of the Al–Cu alloy sample. (*a*, *b*) Grain map of Al matrix together with its completeness map; (*c*) grain boundaries extracted from the Al grain map colored by boundary misorientation angles; (*d*) the segmented Al_2_Cu phase; (*e*, *f*) grain map of the reconstructed Al_2_Cu phase and its completeness map; (*g*) 2D tomographic slice (*Z* = 236) showing Al matrix (gray), eutectic phase (white) and pores (black); and (*h*) an overlay with the Al grain boundary in which two grain boundaries without the eutectic phase, GB_1 and GB_2, are marked.

Fig. 4 ▸ shows the multi-phase grain reconstruction results for the Al–Cu alloy sample, in which the Al matrix and the eutectic Al_2_Cu phase were reconstructed separately using different volume masks that are segmented from the absorption tomography volume. As seen in Figs. 4 ▸(*a*) and 4 ▸(*b*), the Al grain map, consisting of 153 grains with an average diameter of 106.4 ± 71.9 µm, has a nearly random orientation distribution, and the reconstructed voxel completeness is high and close to 1 at the grain centers and decreases towards the grain boundaries. Similar to the Al grain map, the Al_2_Cu grain map, containing 1721 grains with an average diameter of 12.8 ± 10.4 µm, also shows a random orientation distribution, whereas the completeness values are relatively low (∼0.23 on average) because of the much smaller grain sizes [Figs. 4 ▸(*e*) and 4 ▸(*f*)] compared with the Al grain map. High completeness values generally correspond to high confidence in the fidelity of the reconstruction; however, since completeness values systematically decrease for grains with sizes smaller than a certain value, small-grained regions with low values may still contain meaningful shape and orientation information. Regarding the Al_2_Cu grain reconstruction, it was observed that the indexed orientation for the seeding voxels gives completeness values much higher than those obtained with the remaining other orientations, suggesting that most of the reconstructed Al_2_Cu grains, especially the ones with diameters >10 µm (= 831 grains), still have high fidelity and are useful for multi-phase orientation analysis.

To highlight the good correlation between the Al grain boundary and the eutectic phase, 1679 individual Al grain boundaries were extracted [Fig. 4 ▸(*c*)] and compared with the segmented Al_2_Cu phase [Fig. 4 ▸(*d*)]. It is found that not all grain boundaries are decorated with a large amount of the eutectic phase, and some of them are exceptionally ‘clean’ or contain very little. A close look in two dimensions is shown in Figs. 4 ▸(*g*) and 4 ▸(*h*), where a good spatial match is seen between the Al grain boundary and the eutectic phase (difference less than 1–2 pixels). Two grain boundaries showing an absence of the eutectic phase therein are marked in Fig. 4 ▸(*h*). This behavior is considered to be related to the grain-boundary energy which depends on the grain-boundary properties: GB_1 is found to be a small-angle grain boundary (with a misorientation of ∼4.4°) and GB_2 is identified as a Σ15 coincidence grain boundary (CSL, 49.3° misorientation and rotation axis close to [210]), both of which are known to have low grain-boundary energies.

**Figure 5:**
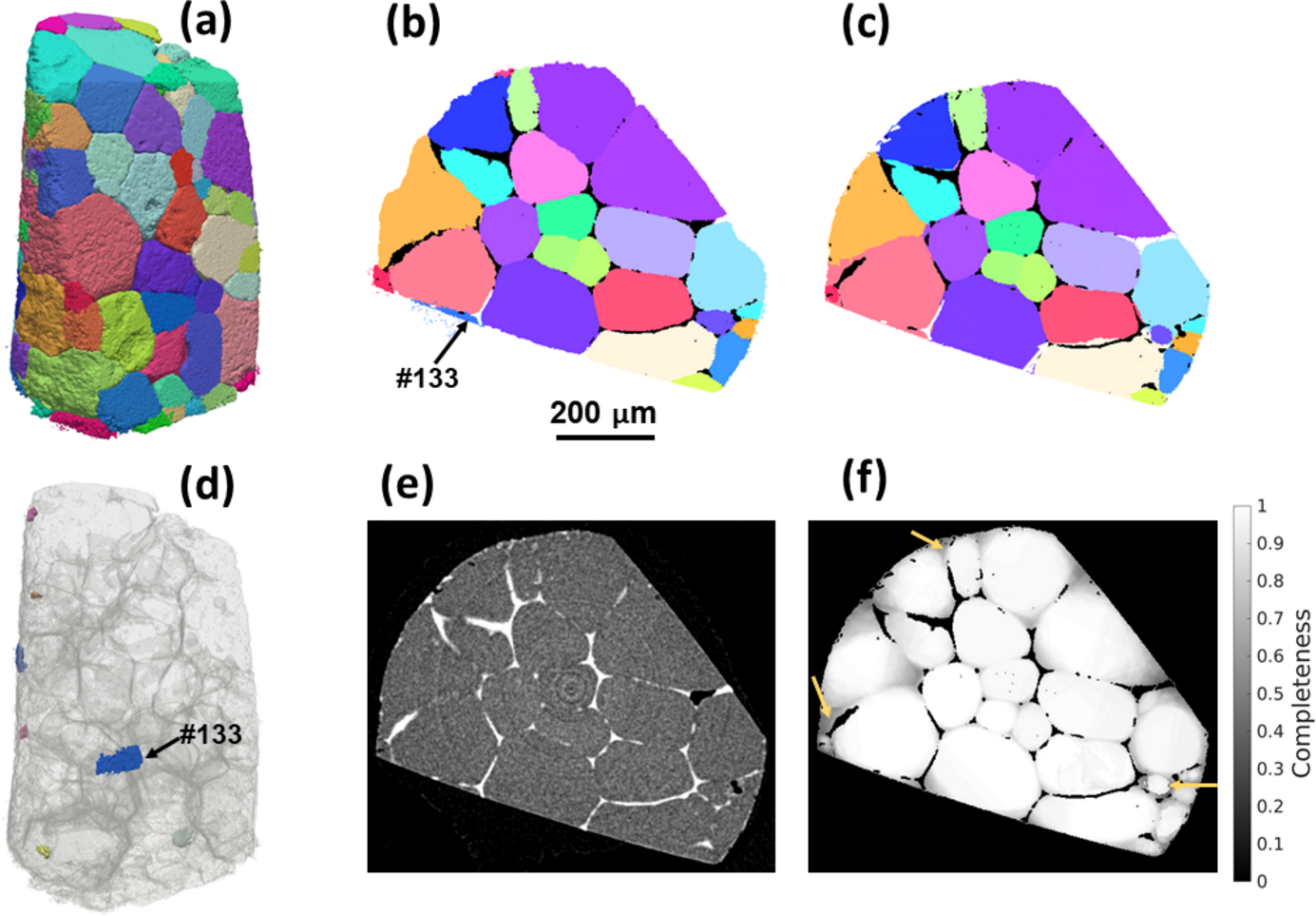
Comparison of grain maps reconstructed with gt-DCT and fwd-DCT. (*a*) gt-DCT reconstructed grain map and (*d*) all grains missed in the reconstruction using fwd-DCT; (*b*) 2D slice (*Z* = 265) from gt-DCT reconstruction and (*e*) the corresponding tomographic slice; (*c*) slice at the same location extracted from fwd-DCT and (*f*) its completeness map. A missed reconstructed grain (#133) is marked in both (*b*) and (*d*). Yellow arrows in (*f*) indicate the regions that have relatively low completeness and show differences from the gt-DCT slice.

Fig. 5 ▸ shows the comparison of the fwd-DCT grain map with the gt-DCT reconstruction using the 6D algorithm, which reconstructed 148 grains with an average diameter of 115.1 ± 62.5 µm. Small differences in grain shapes can be seen in the grain maps by comparing Fig. 5 ▸(*a*) (gt-DCT) and Fig. 4 ▸(*a*) (fwd-DCT). A closer look in two dimensions depicted in Figs. 5 ▸(*b*) and 5 ▸(*c*) shows a good match between them, but also differences in some grain-boundary regions and even missed reconstructed grains, with an example marked as grain #133. With respect to gt-DCT, 7 grains on the sample surface are missed in the fwd-DCT reconstruction [Fig. 5 ▸(*d*)]. To verify the reconstructed grain-boundary position, a corresponding tomographic slice is shown in Fig. 5 ▸(*e*) and a completeness map for fwd-DCT is shown in Fig. 5 ▸(*f*). By looking at these figures together, differences in a few regions close to a grain boundary [lower-completeness regions marked by yellow arrows in Fig. 5 ▸(*f*)] are also evident.

To determine the differences in the grain maps between gt-DCT and fwd-DCT, we first identified the matched and unmatched grains between the two grain maps. When the differences in grain centroid positions and grain average orientations (based on median values of the Rodrigues vectors for all the voxels within the same grain) between any two grains from the two different grain maps are smaller than pre-defined tolerances (Fang *et al.*, 2021 ▸), the two grains are considered as a matched pair. It is found that 143 grains in the gt-DCT map match 148 grains in fwd-DCT, among which 136 grains are uniquely matched and 5 grains in gt-DCT are matched with 11 grains in fwd-DCT (*i.e.* one grain in gt-DCT may be reconstructed as multiple grains in fwd-DCT with similar orientations and spatial position, but sufficiently distinct to be identified as different grains). The remaining 7 grains in either of the two grain maps do not match each other, but they are all relatively small and located on the sample surface and considered as truly existing grains after checking the forward-simulated spots with the experimental projections and cross-validating with the tomography volume (*e.g.* see grain #133 in Fig. 5 ▸).

**Figure 6:**
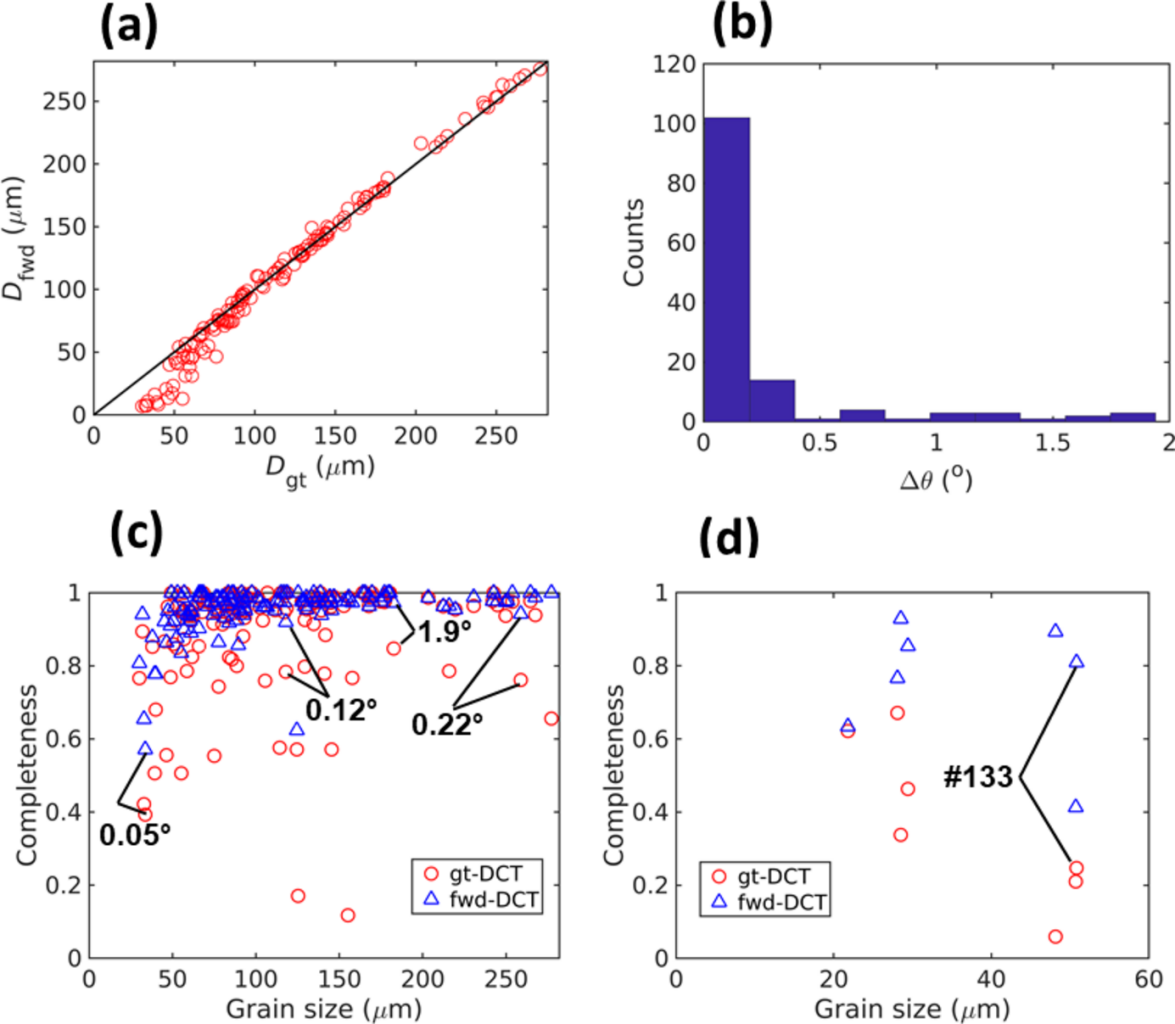
Statistics for the matched and unmatched grains between gt-DCT and fwd-DCT reconstructions. (*a*) Grain size, (*b*) histogram of misorientations and (*c*) completeness values for the 143 matched grains; (*d*) completeness values of the centroid positions for the 7 grains only indexed in gt-DCT. In (*c*) the misorientations between 4 grain pairs are given by marking their respective completeness values in gt-DCT and fwd-DCT. In (*d*) the grain #133 is marked to highlight the higher completeness value claimed by an adjacent, big grain in the fwd-DCT reconstruction. Note that ‘grain size’ in (*c*) and (*d*) corresponds to grains reconstructed in gt-DCT.

We then focus on determining the differences in reconstruction for the matched grains and understanding the reason for mis-reconstructing grains in fwd-DCT. Figs. 6 ▸(*a*) and 6 ▸(*b*) plot the grain size and orientation comparisons, respectively. For grains larger than 50 µm, fwd-DCT shows a very good agreement with gt-DCT, whilst it tends to underestimate the grain size for the smaller ones. This has led to a smaller average grain size overall (see the numbers above). For the reconstructed grain orientations, most of the misorientations are smaller than 0.1°, but there are a few grain pairs that have relatively large misorientations up to ∼2°, resulting in an average misorientation of 0.26° ± 0.41° with a median value of 0.1°. To determine which orientation is more trustworthy, we forward-calculated and plotted the completeness values in Fig. 6 ▸(*c*) for the same grains using the indexed orientations from gt-DCT and fwd-DCT. The completeness values from fwd-DCT are generally higher than those of gt-DCT. For the grains showing large differences in the completeness values, a large orientation difference is also seen, as marked by the misorientation angles in Fig. 6 ▸(*c*).

To examine the reason for missing grains and poorly reconstructed grain boundaries, we have compared the completeness values for these regions using the orientations from gt-DCT (considered as true) and from fwd-DCT. It turned out that the orientation used from the fwd-DCT indeed gives a higher completeness value than the one from gt-DCT (*e.g.* grain #133), which suggests that the poor reconstruction for these regions was caused not by the reconstruction algorithm itself but rather by a lack of diffraction signal from the truly positive grains compared with their neighbors. This is particularly pronounced when the truly positive grain is relatively small and located on the sample surface (diffraction signals often suffer from surface deformation during sample preparation) and there are big grains nearby. Such a tendency, whereby a large grain may compromise the reconstruction accuracy of a small neighboring grain, seems inevitable for fwd-DCT. However, given the completeness map, one could easily identify a badly reconstructed region if it has obviously much lower completeness values than the rest of the same grain, and then filter out the badly reconstructed voxels by applying a threshold on the normalized completeness (Hayashi & Kimura, 2023 ▸) to remedy the reconstruction.

### 4.2. Reconstruction of the Ti alloy sample with 2D box beam and 1D line-focused beam

**Figure 7:**
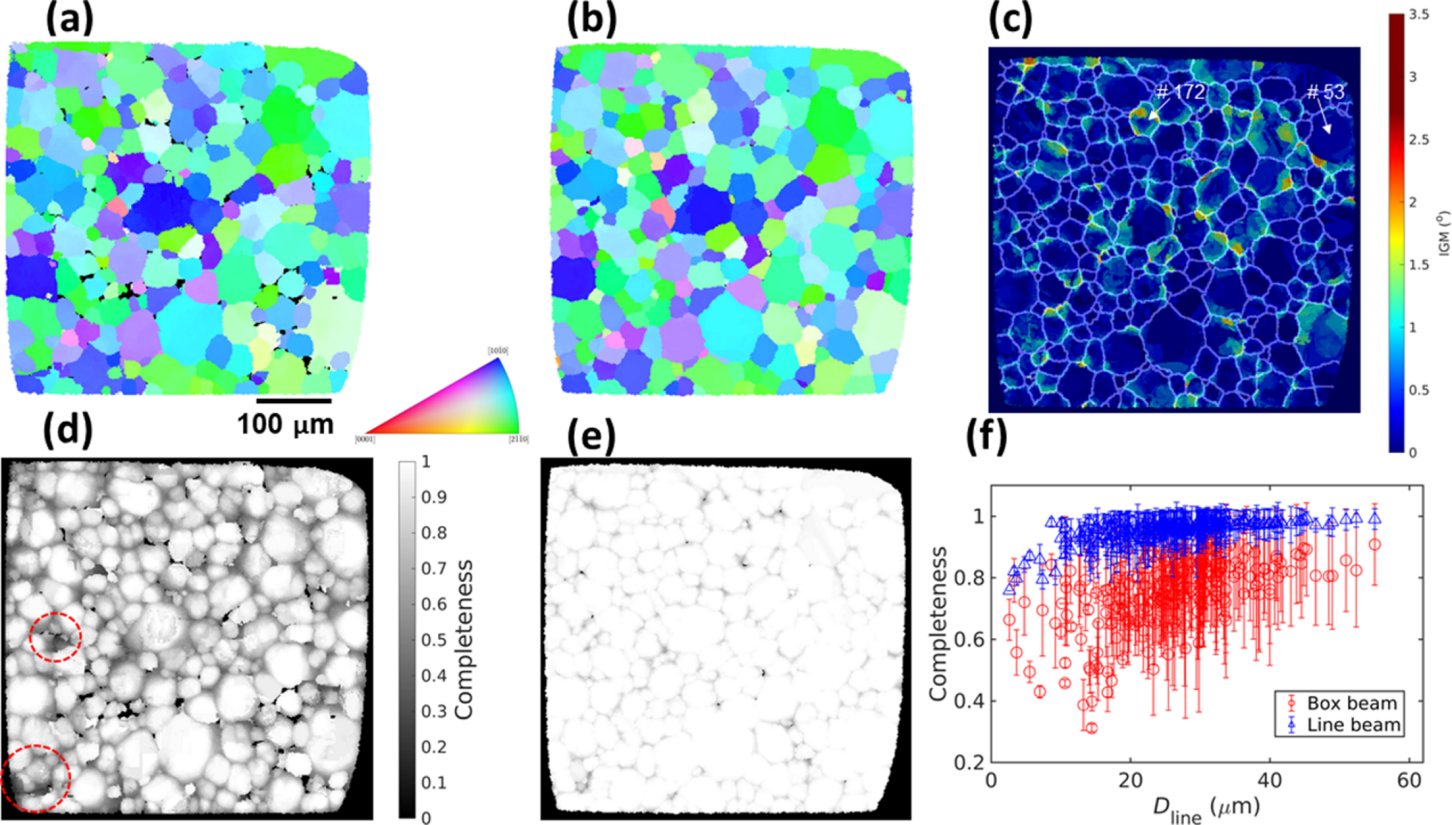
(*a*) Box-beam and (*b*) line-beam grain maps reconstructed with fwd-DCT and their corresponding completeness maps (*d*, *e*); two regions with relatively low completeness values are marked in (*d*). (*c*) Intragranular grain misorientation of the line-beam grain map with the grain boundary shown by white lines and (*f*) comparison of the completeness values for all the grains in the two grain maps.

Fig. 7 ▸ shows the reconstructed fwd-DCT grain maps with significant textures for the deformed α-Ti sample. The two grain maps reconstructed from the box- and line-beam acquisition [Figs. 7 ▸(*a*) and 7 ▸(*b*)] show that the latter can significantly improve the reconstruction, although the two reconstructions are not fundamentally different. The line-beam DCT reconstructs a few more small grains than the box-beam, resulting in 290 grains in total with an average diameter of 24.2 ± 10.1 µm in constrast to 286 grains with an average diameter of 23.3 ± 11.4 µm in the box-beam grain map. Unlike the box-beam grain map, where some grain-boundary voxels remain unindexed, the line-beam DCT fully reconstructed the grain map and shows better consistency in grain shapes [see Figs. 7 ▸(*a*) and 7 ▸(*b*)]. In particular, the regions with relatively low completeness values in box-beam DCT [Fig. 7 ▸(*d*)] now have high completeness values (close to 1), similar to other regions in Fig. 7 ▸(*e*), resulting in better grain shape reconstruction. This is considered true for all the reconstructed grains, as can be seen in Fig. 7 ▸(*f*), where the completeness values of grains in line-beam DCT are much higher than those of their counterparts. The critical grain size showing the transition of the completeness drop decreases from ∼20 µm in box-beam to ∼8 µm in line-beam DCT, indicating a clear potential to improve the detection limit by line-beam DCT.

As each voxel is reconstructed independently with fwd-DCT, each voxel may carry different orientations and thus an IGM (misorientation to the grain mean orientation) map can be computed. The IGM map in Fig. 7 ▸(*c*) shows a significant orientation heterogeneity across all grains. Some of them have a significant gradient of IGM from their centers to their boundaries and the value can reach up to ∼3°.

**Figure 8:**
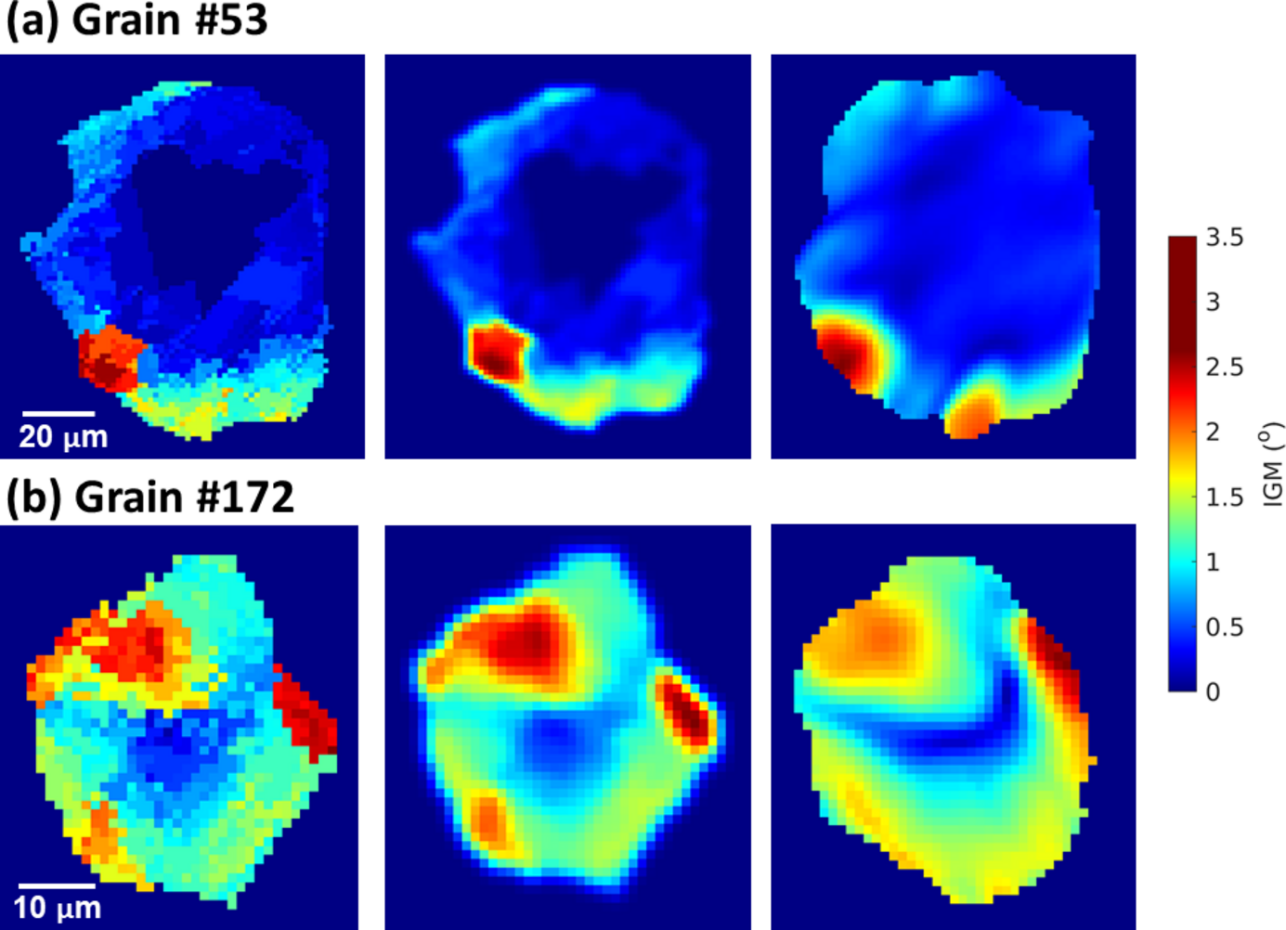
IGM maps for (*a*) grain #53 and (*b*) grain #172. Left: raw reconstruction by fwd-DCT; middle: after applying a Gaussian filter with a sigma value of 1 pixel; right: reconstruction by 6D-DCT algorithm.

We select two grains, #53 with a diameter of 65 µm and #172 with a diameter of 26 µm [see their positions in Fig. 7 ▸(*c*)] from the line-beam DCT, to cross-check their IGM with the 6D-DCT reconstruction, which iteratively forward- and back-projects spots to minimize the errors between forward-simulated and experimentally measured spot intensities. It can be seen in Fig. 8 ▸ that the IGM of both grains agrees with the 6D-DCT reconstruction, with a similar distribution of regions with high IGM and comparable IGM values. The current fwd-DCT reconstruction tends to reconstruct discrete low-angle boundaries and cells, which may be physically realistic. However, it can also be seen that the original IGM reconstructed with fwd-DCT is quite pixelated. In particular, a central region with uniform IGM for grain #53 can be seen in Fig. 8 ▸(*a*). This is because the fwd-DCT only considers the spot position and the same orientation for this central region yields the same maximum completeness of 1. In other words, the fwd-DCT algorithm cannot resolve small variations of local orientation that give rise to the same completeness value. This particular misorientation is determined to be ∼0.1°, which can be considered as the orientation resolution limit for this sample and is postulated to be governed by the step size of the rotation angles during data acquisition.

To smooth the pixelated IGM, a Gaussian filter with a sigma value of 1 pixel (= 1.47 µm) is directly applied. This gives a smoother IGM map that better resembles the IGM reconstructed by the 6D algorithm, which corresponds to a regularized and smoothed version of the orientation field. Nevertheless, small differences in the IGM map and grain shapes can still be observed.

### 4.3. Reconstruction of the fractured Fe–Au alloy sample

**Figure 9:**
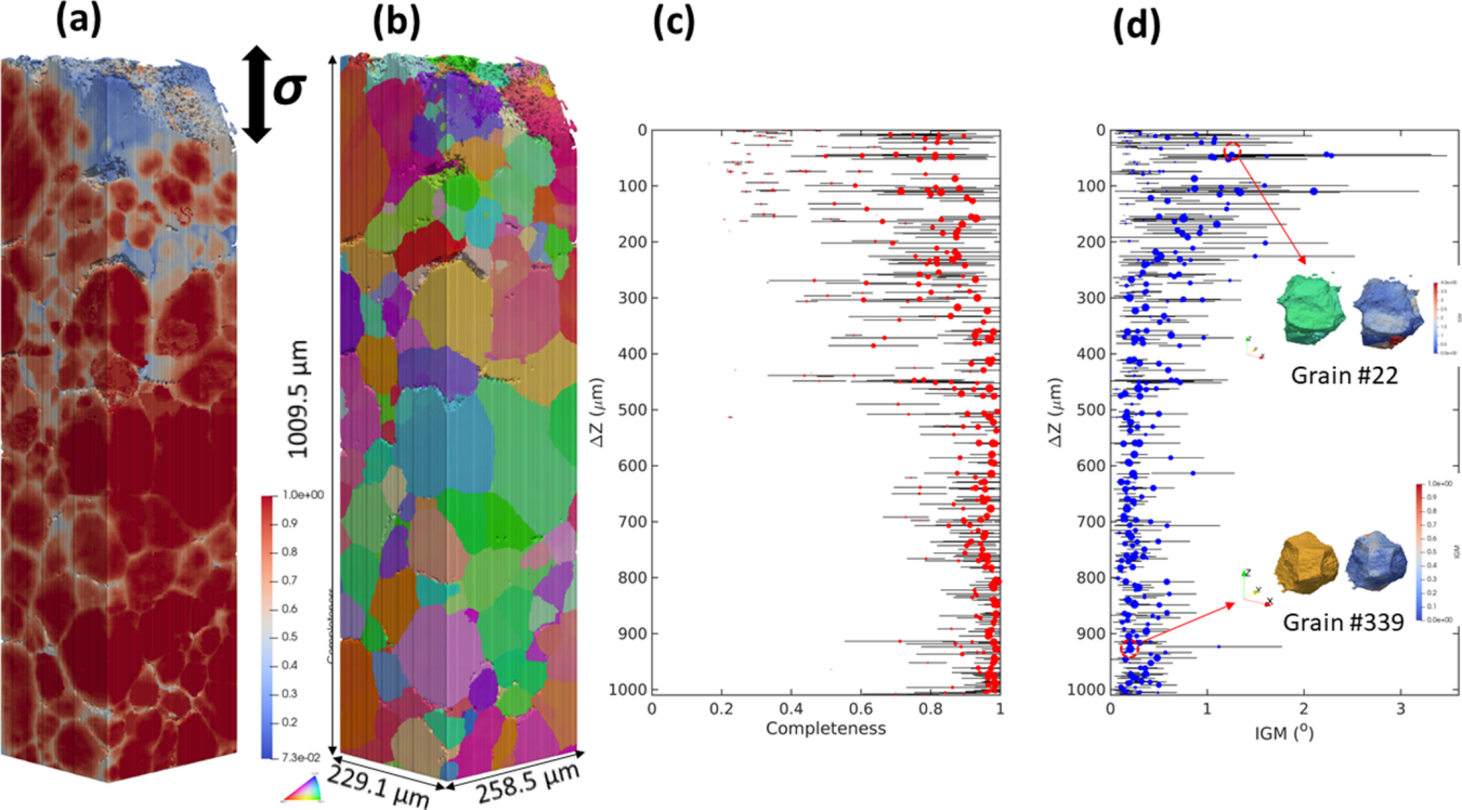
DCT reconstruction of the Fe–Au alloy sample after creep fracture at 550 °C and 80 MPa with the loading direction along the *Z* axis. (*a*) Completeness and (*b*) orientation map in three dimensions; grain positions represented by the distance to the fracture surface (Δ*Z*) plotted as a function of (*c*) completeness value and (*d*) IGM with the error bars corresponding to the standard deviation. Examples of the orientation and IGM map for grain #22 (Δ*Z* = 44.6 µm, equivalent diameter of 79.0 µm and average completeness of 0.813) and #339 (ΔZ = 927.6 µm, equivalent diameter of 135.3 µm and average completeness of 0.988) are shown in (*d*).

Fig. 9 ▸ shows the grain map reconstructed by fwd-DCT for the highly deformed Fe–Au alloy after creep fracture at 550 °C and 80 MPa. The whole grain map was stitched vertically from five subvolumes that were separately reconstructed. As can be seen in Figs. 9 ▸(*a*) and 9 ▸(*b*), the microstructure gradually changes from the top fracture surface to the bottom. The reconstructed grains at the top have lower completeness values [Fig. 9 ▸(*c*)] and higher gradient in intragranular orientations and even show evidence of subgrains as they are more deformed, while the grains located >500 µm away from the fracture surface show much higher completeness and smoother intragranular orientation distribution [Fig. 9 ▸(*d*)]. Such differences are highlighted by the example of two grains located close to and far away from the fracture surface. As shown in Fig. 9 ▸(*d*), grain #22 located close to the fracture surface shows significant orientation spread with an IGM of 1.3 ± 0.7° and the IGM is particularly high close to its grain corners and edges, while grain #339 (located 927.6 µm away) shows a more uniform orientation distribution with an IGM of 0.2 ± 0.1° but a pronounced IGM value can still be seen at the grain surface.

**Figure 10:**
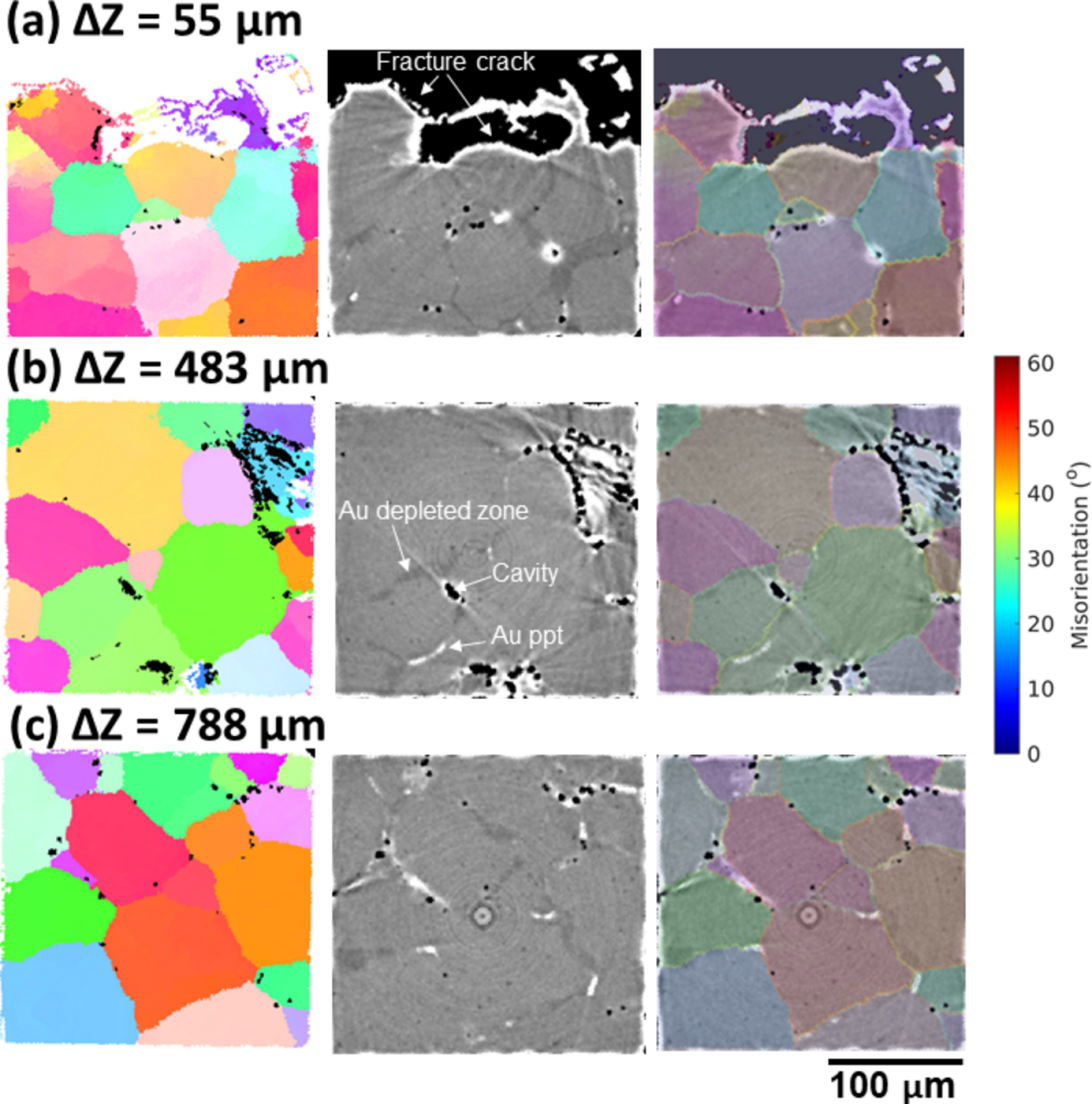
(*a*, *b*, *c*) 2D cross sections perpendicular to the loading direction and sliced at different distances from the top fracture surface (Δ*Z* = 55, 483 and 788 µm, respectively), showing the orientation map (left), tomographic slice (middle) and overlay between the two (right), with grain boundaries colored according to their misorientations. As marked in the tomographic slice, the black, white and dark gray features in the tomographic slices correspond to cavities, Au precipitates and Au-depleted zones, respectively.

To examine the microstructure in detail, three cross sections of the orientation map and the tomographic slice together with the overlay between the two are extracted from different distances to the fracture surface (Δ*Z*) and shown in Fig. 10 ▸. Clearly, the gradient of IGM in slice Δ*Z* = 55 µm is much higher than that in Δ*Z* = 483 and 788 µm as the local deformation is much higher in the region close to the fracture surface. This agrees with the observation from the tomography that creep damage (in the form of cavities and cracks) in large dimensions is mostly found in the region within Δ*Z* < 500 µm, while in the other region the creep cavities mostly remain isolated and relatively small as the grains are less deformed. Additionally, the tomographic results provide a direct proof for checking the quality of the grain reconstruction because the creep cavities and Au precipitates are preferentially formed at the grain boundaries in this sample. The reconstructed grain boundaries agree well with the locations of both creep cavities and gold precipitates, indicating a good grain reconstruction for this highly deformed sample. Interestingly, the gold-depleted zones are always located inside one grain, indicating that Au atoms favor diffusion from one side of the grain boundary to form precipitates at grain boundaries.

## 5. Discussion

### 5.1. Strength of fwd-DCT to overcome the deformation constraint

A longstanding bottleneck in the application of DCT to a large variety of samples is the deformation constraint, especially for the case of box-beam DCT. As the grain is deforming, the diffraction spot becomes distorted and more spread out in both the detector plane and the rotation-angle direction. This causes two problems: (i) lower signal-to-noise ratio and (ii) more spot overlapping, which compromise reliable spot segmentation and successful Friedel-pair matching for the gt-DCT method. The current fwd-DCT method, on the one hand, largely circumvents the spot overlapping problem by exhaustive searching and fitting of the orientation to maximize the completeness for the seed and regional indexing. This implies the diffraction signals (corresponding to a lattice reflection of part of one grain) are directly used rather than diffraction spots (corresponding to a lattice reflection of the entire grain). On the other hand, the lower signal-to-noise ratio problem is also restrained using the LoG spot segmentation method. The challenge of lower signal-to-noise ratio resulting from deformed grains can be further tackled by line-focused DCT, which simultaneously enhances the diffraction intensity and decreases the intensity differences between large and small grains by illuminating their thin sections, instead of their whole volumes as is done in box-beam DCT. As demonstrated by the reconstructions for the moderately deformed Ti sample (see Fig. 7 ▸), the fwd-DCT method can be used for both box-beam and line-focused DCT and shows high flexibility.

The current fwd-DCT method has also been demonstrated to have the capability to reconstruct samples deformed up to ∼10% (see Figs. 9 ▸ and 10 ▸). In practice, samples with an even higher deformation level (*e.g.* ∼18%) have been successfully reconstructed and verified against scanning 3DXRD which has higher resolution and tolerates higher levels of orientation spread. The reconstructed intragranular orientation field has shown reasonable accuracy as compared to the 6D reconstruction (Fig. 8 ▸) and the resolution is mainly limited by the angular step size during data acquisition. However, one has to bear in mind that the quality of the reconstructed grain map might decrease with further increasing deformation. Thus, a completeness map is important for judging the quality – whether a local region is reconstructed correctly or a small grain might be missing in the low-completeness region.

**Figure 11:**
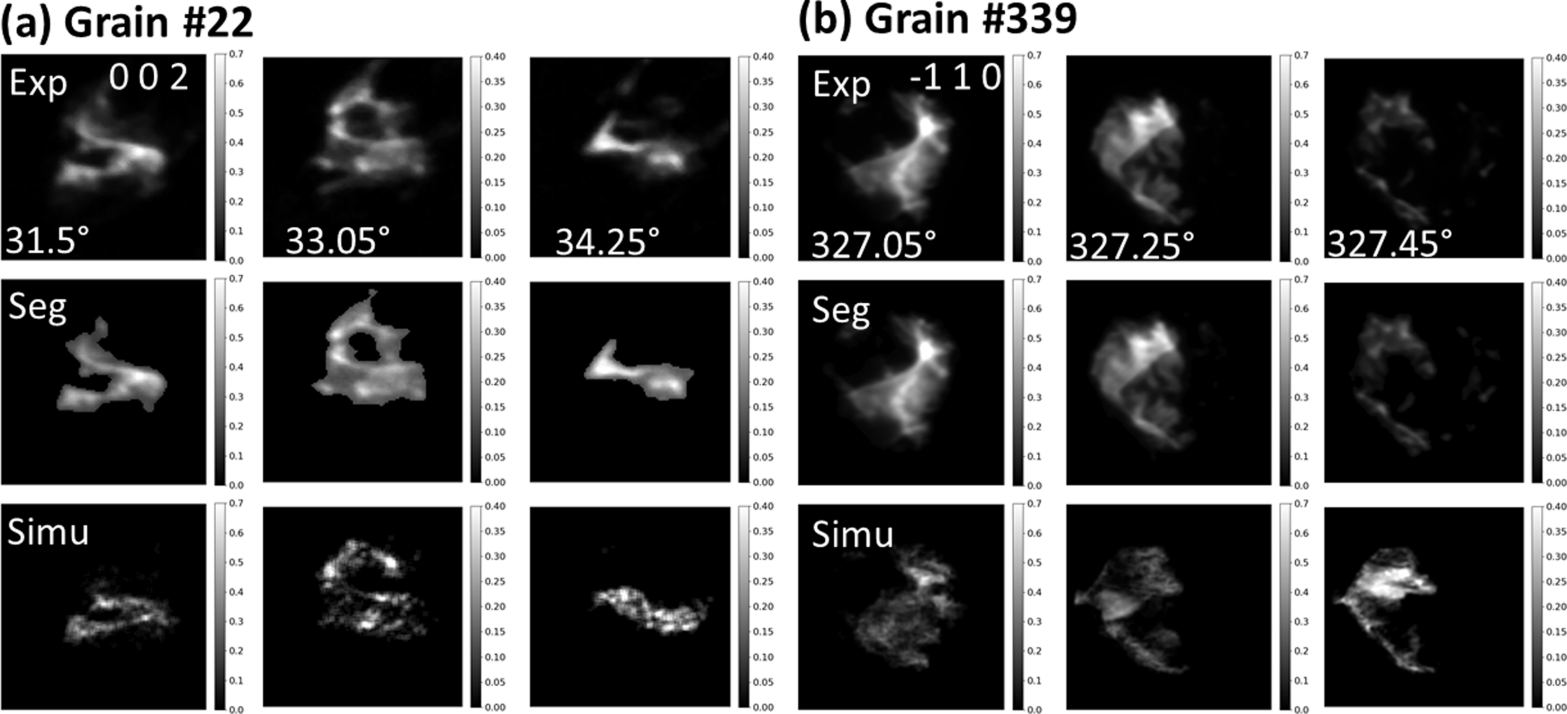
Comparison of simulated spots from reconstructed grains with the experimental spots for (*a*) grain #22 with *hkl* reflection 002 and (*b*) grain #339 with *hkl* reflection 110 in the Fe–Au alloy sample colored by intensities relative to the maximum spot intensity. The first row shows the experimental spots at different rotation angles; the middle row shows the segmented spots used for grain reconstruction; and the bottom row shows the simulated spots from the reconstructed grains.

A common way to validate the fidelity of a grain map is to compare with electron backscatter diffraction [EBSD, see examples given by Syha *et al.* (2013 ▸), Renversade *et al.* (2016 ▸), Menasche *et al.* (2020 ▸) and Sparks *et al.* (2024 ▸)]. However, this often requires careful sample machining, polishing, alignment and post-processing to register the slice or volume between the EBSD and DCT map and is therefore not straightforward. In addition to comparing with other X-ray diffraction techniques [such as high-resolution scanning 3DXRD as mentioned above (Henningsson *et al.*, 2024 ▸)] and given the capability to reconstruct deformed samples, another effective and straightforward way is to compare the forward-simulated spots from the reconstructed grains with the experimental spots. As shown in Fig. 11 ▸, we have computed two spots from two different reconstructed grains (see Fig. 9 ▸ for the grain information) which have different deformation levels. A reasonably good agreement of the spot shapes and intensity distribution between the simulated and experimental ones is observed, although small differences in the details can also be seen. This suggests the reconstruction is not perfect yet. As the reconstruction relies on segmented spots, imperfect spot segmentation is one of the primary error sources. This is more pronounced in the more deformed grain #22 compared with grain #339, as the very weak signals are not segmented. Nevertheless, the current fwd-DCT method only uses the position of the diffraction signals for reconstruction, like all the other nf-HEDM reconstruction software, and has not exploited the full information to include the spot intensity for further refinement. Ultimately, the forward model would have to consider elastic distortions of the crystal lattice – an outstanding endeavor for which first promising strategies have been proposed by Shen *et al.* (2020 ▸) and Reischig & Ludwig (2020 ▸).

### 5.2. Weakness of fwd-DCT compared with gt-DCT

Although the current fwd-DCT method has the advantage of overcoming the deformation constraint and is easy to use with little adjustment of the reconstruction parameters, it comes at the cost of a significant increase in the reconstruction time. The fwd-DCT method has to visit as many seeding points as possible, especially around grain boundaries, to reconstruct a good grain map (see Fig. 3 ▸). Normally the number of seeding points is proportional to the number of grains and the size of the sample volume. To give a rough comparison, testing 10000 seeding points (including seeding and regional indexing) using one GPU machine requires ∼24 h, whilst gt-DCT takes about ∼0.5 h to reconstruct a sample with ∼1000 grains. To reduce the computing time, we have implemented the slice-by-slice (in all three orthogonal planes) reconstruction with fwd-DCT by using multiple GPU machines simultaneously. We also acknowledge the existence of some redundancies in the seed–regional indexing algorithm. In future development, we will try to remove the unnecessary calculations to improve computational efficiency, whilst we continue to ensure an autonomous reconstruction without the need to tune reconstruction parameters.

Another weakness is that fwd-DCT has the tendency to miss or poorly reconstruct a small grain that is located near a big grain because the latter could carry its higher completeness values to the actual space of the small grain. For gt-DCT, as long as the minimum required number of Friedel pairs can be assigned to a small grain (usually ≥4), it can be indexed and the shape reconstruction is less affected by the nearby big grains because it takes into account spot intensities for reconstruction. To effectively circumvent this issue with fwd-DCT, three approaches can be employed. The first is to apply an adaptive percentage value for the intensity cut-off in the LoG spot segmentation in relation to the detector point spread function, instead of using a constant percentage (typically 0.08–0.16) as used at present, to suppress the over-segmentation of the stronger spots from the larger grains. The second is to employ a deconvolution algorithm with a variable point spread function (*e.g.* Baddeley *et al.*, 2006 ▸) for the raw projection images. The third approach is to use line-beam DCT to decrease the bias in the completeness induced by the grain size, as has been demonstrated by the comparison between box-Ti and line-Ti reconstructions (Fig. 7 ▸).

### 5.3. Combined use of fwd-DCT and gt-DCT methods

**Figure 12:**
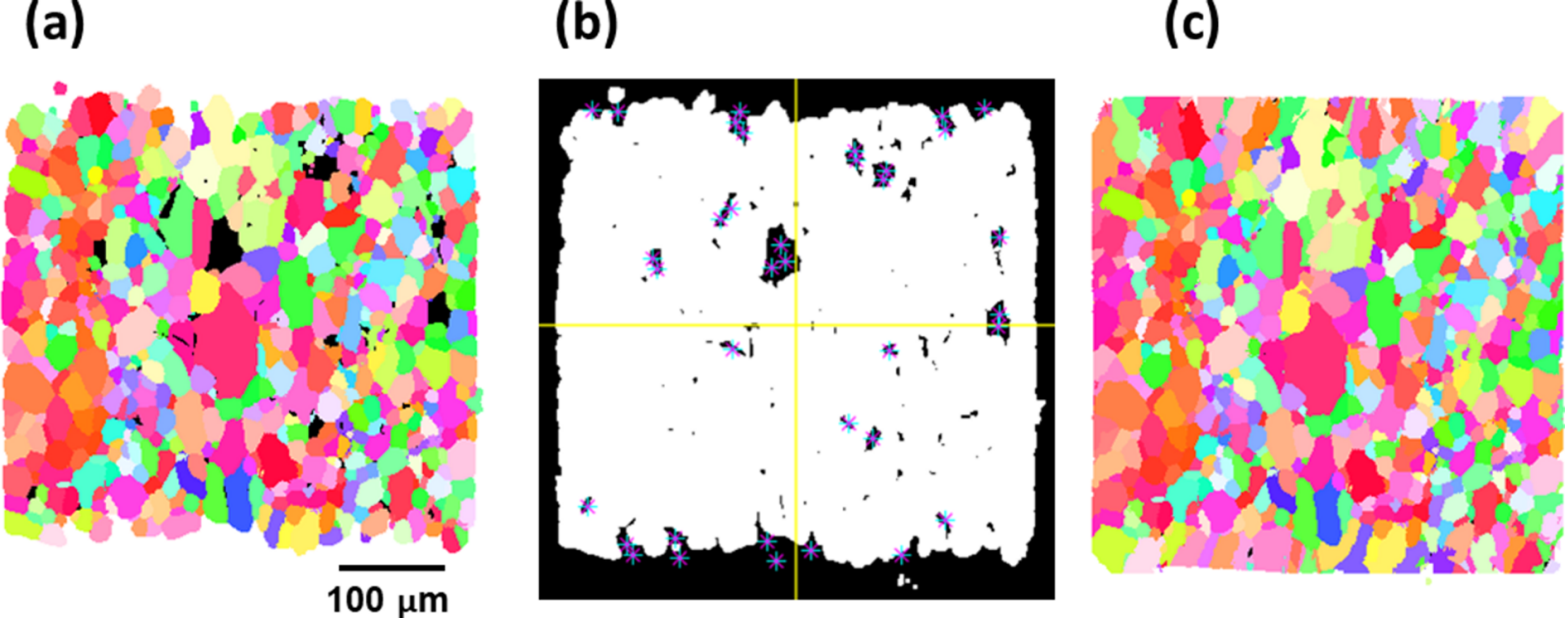
Finding missing grains in a grain map by fwd-DCT illustrated by a 2D cross section. (*a*) Original grain map reconstructed by gt-DCT, (*b*) interactively placing the seeding point on a mask and (*c*) the final reconstructed grain map.

As discussed above, fwd-DCT and gt-DCT have their own advantages to process DCT reconstructions. One can efficiently combine the two methods for grain mapping. As the Friedel-pair matching in gt-DCT is very efficient in fitting the geometry, fwd-DCT uses this fitted geometry by default and therefore it is not required to fit its own geometry by reconstructing a first grain map (Fang *et al.*, 2023 ▸). Conversely, it is not uncommon to have holes, corresponding to missing grains, in the grain map reconstructed with gt-DCT. The reason for the missing grains is, however, sometimes not trivial to resolve (lack of matched Friedel pairs, wrong matching or deformed grains *etc*). Using fwd-DCT, finding the missing grains can be efficiently done, thus rendering a full grain map quickly. As shown in Fig. 12 ▸(*a*), the original grain map reconstructed by the gt-DCT method has quite a few missing holes. To find the missing grains, one can place the seeding points in the holes [Fig. 12 ▸(*b*)] and run the reconstruction with fwd-DCT. It can be seen in Fig. 12 ▸(*c*) that the missing grains have been nicely reconstructed in the final grain map.

To fully exploit the spot intensity for grain reconstruction, the 6D-DCT reconstruction algorithm (Viganò *et al.*, 2016 ▸; Viganò & Ludwig, 2020 ▸) can be used as a further refinement for the intragranular orientation reconstruction for individual grains. As the resolution of the IGM map reconstructed from fwd-DCT is limited by the rotation step size, the resolution may not be sufficient to resolve fine features such as slip bands. Therefore, incorporating the 6D reconstruction algorithm to include the intensity information for further refining the intragranular reconstruction is attractive.

### 5.4. Outlook for future developments

The currently developed fwd-DCT method opens up more possibilities for reconstructing deformed samples. By combining it with the use of gt-DCT, we envisage DCT will remain an appealing technique for resolving a 3D grain map in a short timescale compared with layer-by-layer nf-HEDM or scanning 3DXRD, thereby showing great potential for *in situ* grain mapping as a function of stimuli.

Compared with far-field techniques such as scanning 3DXRD, near-field DCT has its intrinsic limitations – it is much less sensitive in detecting small grains and elastic strains and has very limited space for mounting sample environments. Therefore, integrating the near-field and far-field techniques as a standard routine, both hardware and software, is highly desirable to realize multi-scale grain and strain mapping.

## 6. Conclusions

A forward-model-based reconstruction method has been developed for synchrotron diffraction contrast tomography. Benchmark reconstructions have been performed on fully recrystallized Al–Cu alloy, moderately deformed α-Ti alloy and highly deformed Fe–Au alloy samples. These reconstructions demonstrate that the current fwd-DCT method can overcome the longstanding bottleneck of deformation constraint and has great potential in reconstructing samples deformed up to ∼10% plastic strain. In addition, the novel method can reconstruct intragranular orientations reasonably well and is suitable for multi-phase materials with both box-beam and line-beam acquisition geometries. In combination with the conventional Friedel-pair-matching-based method and the 6D orientation reconstruction framework, it shows great potential in expanding the application of the DCT method to a broad range of scientific cases.

## Figures and Tables

**Table 1:** The number of discretized orientations (*N*_global_OR_) for different crystal structures and the average minimum inter-distance between orientations with the standard deviation The discretization methods include hyper-spherical orientation sampling (Larsen & Schmidt, 2017 ▸) for the cubic structure and mesh gridding on Rodrigues–Frank space (He & Jonas, 2007 ▸) for other crystal systems.

Crystal structure	*N*_global_OR_	Average distance (°)	Method
Cubic	262144 or 32768	1.3 ± 0.1 or 2.6 ± 0.2	Hyper-spherical sampling
Hexagonal	88200	2.2 ± 0.5	Mesh gridding
Tetragonal	137200	2.1 ± 0.4	Mesh gridding
Trigonal	280000	1.2 ± 0.1	Mesh gridding
Orthorhombic	343000	1.6 ± 0.1	Mesh gridding
Monoclinic	374530	2.7 ± 0.1	Mesh gridding
Triclinic	743451	2.7 ± 0.1	Mesh gridding

**Table 2:** Experimental parameters of DCT scans

Scan name	Beam size (width × height) (µm^2^)	*L*_sd_ (mm)	Effective pixel size (µm)	*t*_exp_ (s)	No. of projections	No. of vertical scans
AlCu	850 × 1000	7.25	1.63	0.1	3600	1
Box-Ti	900 × 30	8.94	1.47	0.05	7200	1
Line-Ti	900 × 2	9.24	1.47	0.1	3600	1
FeAu	550 × 250	5.26	1.22	0.1	7200	5

## Appendix A Formulation of forward calculation

Given the laboratory coordinate system shown in Fig. 1 ▸, we define the origin (0, 0, 0) as the middle intersection of the rotation axis and the incident beam. During the projection acquisition while the sample is rotating, the scattering vector ***G***_s_ in the sample coordinate system for an (*hkl*) plane of a grain fulfilling the Bragg diffraction condition can be expressed as

where ***U*** is a matrix transforming a Cartesian crystal system to the sample system, *i.e.* crystal orientation, ***B*** is a matrix transforming reciprocal space to the Cartesian crystal system determined by the crystal unit cell and . More details about the formulation of these transformation matrices are presented by Poulsen (2004 ▸).

For diffraction of a monochromatic beam with a wavelength of λ, the diffraction angle θ can be calculated by the following equation:

where  stands for the length of the diffraction vector. In order to calculate the diffraction vector in the laboratory coordinate system, ***G***_lab_, we have to calculate the rotation angle ω over which the diffraction is expected to occur. According to Schmidt (2014 ▸), ω should fulfill the following two equations:

where the parameters *a*, *b*, *c*, *d* and *e* are calculated as follows:

Combining equations (1)–(9), the solution for ω can thus be derived and there may be 0, 1 or 2 solutions in the range of 0 and 2π. If there exists any solution for ω, ***G***_lab_ can be derived by

where

is a transformation matrix to convert the sample system to the laboratory system.

Once ***G***_lab_ is derived, for a given sample position  in the laboratory system [converted from the position in the sample system by ], the spot position  on an ideal detector (with zero offsets and tilts relative to the incident beam) at ω rotation can be calculated by

where *L*_diff_ is calculated by

with *L*_sd_ denoting the sample-to-detector distance.

To derive the spot position  on a non-ideal detector, we define the detector offsets as  and tilts denoted by a rotation matrix ***R***_det_, which can be calculated as  with the rotation matrices expressed by the tilt angles  about the *x*, *y*, *z* axes, respectively, as follows:

We then rewrite the unit vector of the diffracted beam as

Following Fang *et al.* (2022 ▸), the intersected spot position  should fulfill

where *t* can be solved as

with *R*_*ij*_ (*i*, *j* = 1, 2 or 3) denoting an element of ***R***_det_. Thus, the intersection position can be finally derived as follows:

Therefore, for a given sample position  with an orientation denoted by the ***U*** matrix and a unit cell to determine the ***B*** matrix, the expected spot position on the detector plane is . In the current forward-model reconstruction, the ***B*** matrix is considered as a constant matrix for the same phase and only the orientation is solved by maximizing the completeness. Once the orientation solution is derived, a refinement of the ***B*** matrix can in principle be performed, but this is not in the scope of this work.

For computing forward-simulated projections using a synchrotron monochromatic beam, the spot intensity *I*_spot_ from an illuminated volume element *V*_element_ can be calculated by (Als-Nielsen & McMorrow, 2011 ▸)

where  is the attenuation factor due to sample absorption for photons with energy *E*_*hkl*_,  is the incident flux of the beam, *r*_0_ is the Thomson scattering length and amounts to  = 2.82 × 10^−15^ m,  is the structure factor of the *hkl* reflection, *L*_g_ is the Lorentz factor calculated by  with η expressing the azimuthal angle, *P*_0_ is the polarization factor and is taken as 0.95, *t*_exp_ is the exposure time per projection, and *v* is the volume of the unit cell. The detector quantum efficiency is not included in this equation as it is expected to be constant for all reflections with the monochromatic beam. More details can be found in the work of Fang *et al.* (2020 ▸).
